# eNAMPT-IFITM3 axis links neuroinflammation to γ-secretase activation

**DOI:** 10.1016/j.isci.2026.117496

**Published:** 2026-09-12

**Authors:** Junho Hyun, Sunmin Jung, Yoona Noh, Yeonsu Cho, Takaomi C. Saido, Jihoon Nah, Ki Woo Kim, Yong-Keun Jung

**Affiliations:** 1 School of Biological Science, Seoul National University, Seoul 08826, Korea; 2 Division of Physiology, Yonsei University College of Dentistry, Seoul 03722, Korea; 3 Advanced-Basic-Convergence Research Institute, Chungbuk National University, Cheongju 28644, Korea; 4 Department of Biochemistry, Chungbuk National University, Cheongju 28644, Korea; 5 Laboratory for Proteolytic Neuroscience, RIKEN Brain Science Institute, Wako-Shi, Japan

**Keywords:** Alzheimer’s disease, γ-secretase, NAMPT, IFITM3, amyloid beta

## Abstract

Amyloid-β (Aβ) accumulation and neuroinflammation are central features of Alzheimer’s disease (AD), yet the molecular link connecting inflammatory signaling to γ-secretase activation remains poorly understood. Here, using a cell-based gain-of-function screen with a cDNA library, we identify nicotinamide phosphoribosyltransferase (NAMPT) as a positive regulator of γ-secretase activity. We demonstrate that extracellular NAMPT (eNAMPT) acts as a paracrine factor binding to cell-surface interferon-induced transmembrane protein 3 (IFITM3), enhancing its protein stability and promoting γ-secretase complex assembly to heighten amyloidogenic processing, independently of its NAD+ biosynthetic function. Pathologically, NAMPT levels are elevated in patients with AD and mouse models. Furthermore, systemic inflammation induced by lipopolysaccharide (LPS) enhances NAMPT expression and promotes the NAMPT-IFITM3 interaction and γ-secretase complex assembly *in vivo*, whereas NAMPT neutralization with an antibody suppresses LPS-induced *γ*-secretase activation. These findings establish the eNAMPT-IFITM3 axis as a key molecular link bridging neuroinflammation and γ-secretase-mediated AD pathology.

## Highlights

• eNAMPT preferentially enhances APP-related γ-secretase processing
• eNAMPT binds the IFITM3 C-terminus to increase the IFITM3-associated γ-secretase complex
• Neutralizing eNAMPT suppresses the LPS-induced γ-secretase activation in AD mice

## Graphical abstract

**Figure:**
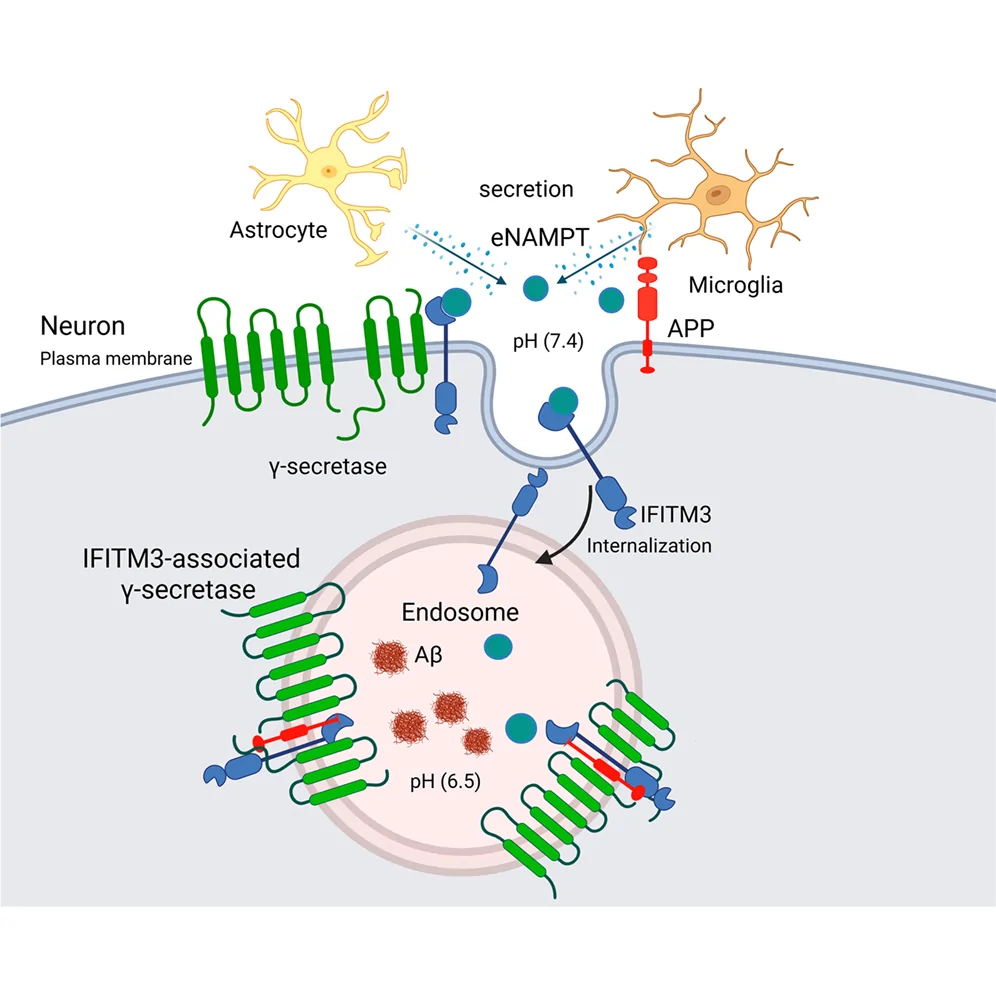

## Teaser

Immunology; Neuroscience

## Introduction

Amyloid plaques, characterized by the deposition of neurotoxic amyloid-β (Aβ) peptides that aggregate into soluble oligomers and insoluble extracellular plaques, are defining pathological hallmarks of Alzheimer’s disease (AD).^1^^,^^2^ Aβ generation is widely recognized as a central event in disease initiation and occurs through the sequential proteolytic cleavage of the APP by β-secretase (BACE) and γ-secretase.^3^ The γ-secretase is a terminal enzyme in the amyloidogenic pathway, and its overall catalytic activity is thus tightly coupled to the total production yield of Aβ. The γ-secretase is a large multiprotein intramembrane complex of approximately 440 kDa, composed of four essential core subunits; presenilin (PS1 or PS2), the catalytic component; nicastrin (NCT) for substrate recognition; anterior pharynx-defective 1 (APH1), a structural scaffold for complex assembly; and presenilin enhancer 2 (PEN2), which facilitates the endoproteolytic cleavage of presenilin to its active form.^4^ This complex catalyzes the cleavage of APP, determining the overall level of Aβ peptides generated.^5^ In addition, γ-secretase cleaves numerous substrates, most notably Notch, highlighting the critical importance of identifying modulators that specifically regulate APP-cleaving activity.^6^^,^^7^ Recent studies underscore that γ-secretase activity is regulated by internal and external modulators, particularly within the endolysosomal compartments.^8^^,^^9^ While various modulators have been identified,^10^^,^^11^^,^^12^^,^^13^^,^^14^ the precise mechanisms dictating γ-secretase activity under AD pathology, such as inflammation, remain poorly understood.

Nicotinamide phosphoribosyltransferase (NAMPT), the rate-limiting enzyme in the NAD+ salvage pathway, converts nicotinamide (NAM) to NAM mononucleotide (NMN), a precursor for NAD+ synthesis for maintaining cellular energy homeostasis, DNA repair, and redox balance.^15^^,^^16^^,^^17^^,^^18^^,^^19^ Beyond its enzymatic functions, NAMPT also acts as a pro-inflammatory cytokine-like molecule, often referred to as pre-B-cell colony-enhancing factor (PBEF) or visfatin, underscoring its dual functionality in metabolic and inflammatory processes.^20^^,^^21^^,^^22^ NAMPT’s involvement in inflammation is increasingly recognized across various disease contexts.^23^ In acute lung injury and rheumatoid arthritis, NAMPT amplifies inflammatory signaling.^24^^,^^25^^,^^26^ In metabolic disorders, such as obesity and type 2 diabetes, NAMPT levels are elevated and contribute to chronic low-grade inflammation.^27^^,^^28^^,^^29^^,^^30^ These findings highlight NAMPT as a key player at the intersection of metabolic dysregulation and inflammatory signaling. In the central nervous system, NAMPT is expressed across various cell types and is upregulated in response to inflammatory stimuli, such as lipopolysaccharide (LPS), further linking NAMPT to neuroinflammation.^31^^,^^32^ While neuroinflammation, a driver of AD pathology,^33^ activates γ-secretase, the identity of the secreted inflammatory factor released by reactive glial cells and acting on neighboring neurons for γ-secretase activation remains a critical missing link.^34^^,^^35^^,^^36^ Recently, interferon-induced transmembrane protein 3 (IFITM3), an endolysosomal protein, has emerged as a key γ-secretase regulator, which interacts with γ-secretase to promote amyloidogenic processing.^34^ However, the upstream regulation of IFITM3 function or association with γ-secretase during neuroinflammation is not clear.

To better understand the molecular connection between inflammation and γ-secretase, we have screened a cDNA expression library encoding 1,800 human secretory proteins using a cell-based assay of γ-secretase and isolated NAMPT. In the present study, we demonstrate that eNAMPT, secreted by astrocytes, acts as a molecular messenger that specifically engages IFITM3 on the neuronal cell surface to enhance γ-secretase activity and Aβ production. Our findings establish the NAMPT-IFITM3 axis as a crucial molecular connection, solving the long-standing question of how neuroinflammation is coupled to γ-secretase activation.

## Results

### NAMPT increases γ-secretase activity with a preference for APP processing

**Figure 1:**
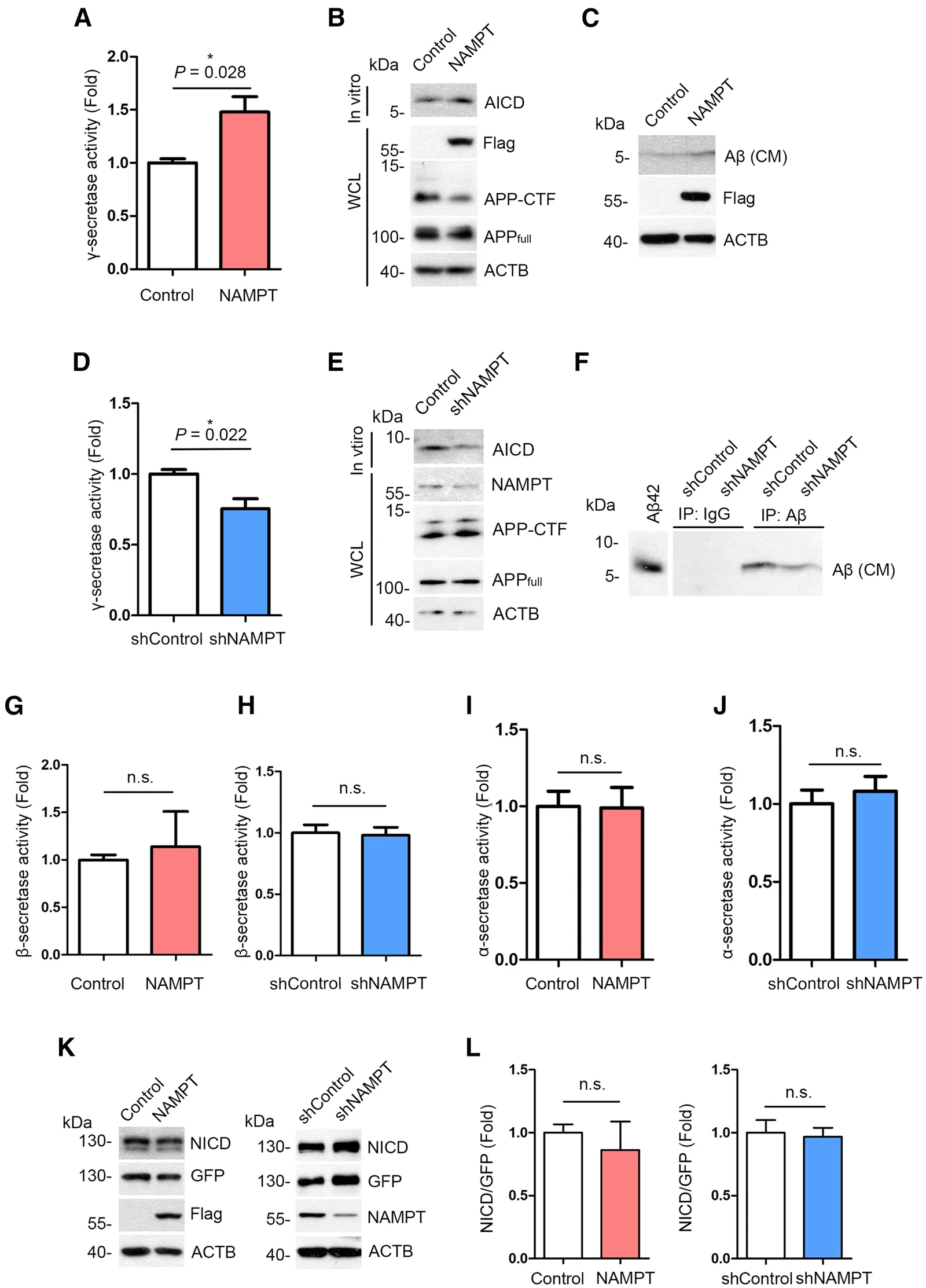
NAMPT promotes γ-secretase activity with a preference for APP processing (A–C) NAMPT-FLAG overexpression enhances γ-secretase activity and Aβ production. HEK293T (A) and SH-SY5Y/APPsw (B and C) cells overexpressing NAMPT-FLAG were assessed for γ-secretase activity with a C99-GVP luciferase reporter assay (A), for AICD levels by *in vitro* AICD generation assay (B), and for secreted Aβ levels in the conditioned medium by immunoprecipitation (IP) assay using 6E10 or anti-Aβ antibody (C). WCL, whole cell lysates. (D–F) Knockdown of NAMPT expression reduces γ-secretase activity. HEK293T (D), SH-SY5Y/APPsl-PS1ΔE9 (E), and SH-SY5Y/APPsw (F) cells silencing NAMPT expression with shRNA were assessed for γ-secretase as described in (A–C). WCL, whole cell lysates. (G–J) NAMPT levels do not affect the activities of β- and α-secretase. HEK293T cells overexpressing (G) or silencing (H) NAMPT were assessed for β-secretase activity with a Bace1 reporter assay (G and H). Culture medium of CHO-7PA2 cells overexpressing (I) or silencing (J) NAMPT was examined for α-secretase activity by measuring sAPPα with western blotting. n.s., not significant. (K and L) NAMPT does not affect Notch cleavage. HEK293T cells were transfected with NotchΔE-GFP and either NAMPT (K, left) or NAMPT shRNA (K, right) for 24 h and analyzed with western blotting. The signals of NICD on the blots were quantified using ImageJ (L). n.s.; not significant. Bars represent mean ± SEM (A, *n* = 4; D, *n* = 5; G–J, *n* = 3; L, *n* = 4), ∗*p* < 0.05, n.s., not significant (Student’s *t* test).

To identify positive regulators of γ-secretase, we have screened a cDNA expression library encoding 1,800 secretory proteins with a γ-secretase reporter assay which utilizes pC99-TetOn and pTRE-GFP, as previously described.^37^ Through an unbiased genome-wide gain of functional screen, we have isolated several positive cDNA clones that enhanced the reporter activity reflecting γ-secretase activity in HEK293T cells.^10^^,^^11^^,^^12^ Among them, NAMPT was potent to stimulate γ-secretase after transient overexpression in the cell-based assay. Using C99-GVP luciferase reporter assay, we confirmed that NAMPT overexpression significantly enhanced γ-secretase activity by 1.5-fold compared to control cells, while NAMPT knockdown significantly reduced it (Figures 1A and 1D). Consistently, *in vitro* APP intracellular domain (AICD) generation assays in SH-SY5Y/APPsw and SH-SY5Y/APPsl-PS1ΔE9 cells revealed that NAMPT overexpression enhanced APP processing to increase the levels of AICD, a product of APP processing by γ-secretase, while NAMPT knockdown reduced it (Figures 1B and 1E). Accordingly, NAMPT overexpression and knockdown affected the levels of secreted Aβ in culture medium of SH-SY5Y/APPsw cells (Figures 1C and 1F). These findings suggest that NAMPT is a positive activator of γ-secretase and Aβ generation.

We next assessed the effects of NAMPT on β- and α-secretase activities using corresponding reporter assays. Intriguingly, unlike its stimulatory effect on γ-secretase, the activities of β- and α-secretases were not significantly altered by NAMPT overexpression (Figures 1G and 1I) or knockdown (Figures 1H and 1J). Thus, NAMPT modulates γ-secretase without influencing the activities of β- and α-secretases. In addition, because NAMPT stimulates γ-secretase activity and APP processing, we evaluated whether NAMPT also affects Notch cleavage by analyzing the levels of Notch intracellular domain (NICD), a product of Notch processing by γ-secretase. The results of western blot analysis in HEK293T cells expressing NotchΔE-GFP revealed that the levels of NICD generated by γ-secretase were not altered by NAMPT expression levels (Figures 1K and 1L). NAMPT levels following its overexpression and knockdown in the respective cells were confirmed by western blotting (Figures 1B–1E and 1K). These results indicate that NAMPT selectively activates γ-secretase to increase APP processing, rather than Notch cleavage.

### γ-Secretase activity in neurons increases by glial cell-derived eNAMPT independent of its enzyme activity

To investigate whether the enzyme activity of NAMPT is associated with its stimulatory effect on γ-secretase, we utilized the NAMPT inhibitor FK866.^38^ With AICD generation assay, we found that treatment of HEK293T/APPsw cells with FK866 did not affect γ-secretase activity compared to untreated control (Figures S1A and S1B). In addition, treatment of HEK293T/APPsw membrane fractions with NAD^+^ did not alter γ-secretase activity either. We also tested the effects of the NAMPT dominant-negative (DN) mutant (H247A), which is unable to convert NAM to NMN.^39^^,^^40^ The results of the C99-GVP luciferase reporter assay revealed that expression of NAMPT DN mutant significantly increased γ-secretase activity (Figure S1C). On the other hand, this stimulatory effect was impaired by NAMPT S200D mutant, which loses the ability to form NAMPT dimer and thereby enzyme activity.^41^ In addition, unlike NAMPT wild type (WT) and H247A mutant, NAMPT S200D mutant was not detected in the conditioned medium (CM) of HEK293T cells (Figure S1D). These studies suggest that NAMPT stimulates γ-secretase activity through eNAMPT, rather than its enzymatic role in producing NAD^+^.

**Figure 2:**
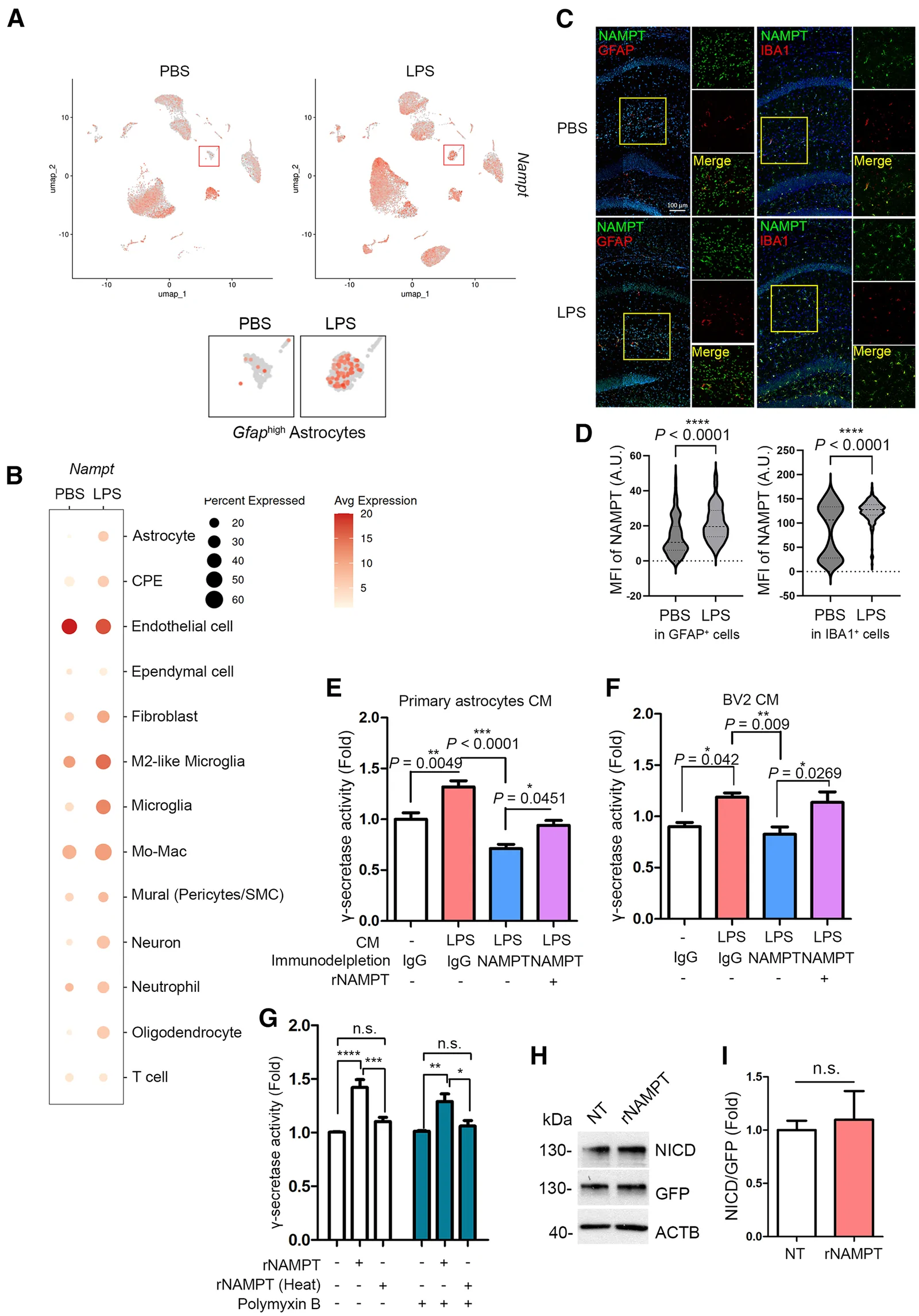
NAMPT secreted from astrocyte and microglia upon LPS treatment enhances γ-secretase activity in neurons (A) Uniform manifold approximation and projection (UMAP) feature plots illustrating cell-type-specific distribution and levels of *Nampt* transcripts in PBS- and LPS-treated (LPS × 2, daily 0.8 mg/kg, i.p.) mouse brain from GEO dataset GSE263094 (upper). An enlarged view of the *Gfap* high astrocyte cluster is shown to highlight the prominent increase of *Nampt* expression in astrocytes following repeated systemic LPS administration (lower). (B) Dot plot showing *Nampt* expression across annotated brain cell populations under PBS- and LPS- treated conditions. Dot size represents the fraction of *Nampt*-expressing cells within each cluster and color intensity represents average expression level. (C) Representative images of brain sections immunostained for NAMPT together with cell-type markers, including GFAP for astrocytes and IBA1 for microglia. Mice were i.p. injected with PBS or 0.5 mg/kg LPS daily for two consecutive days and brain sections were prepared 24 h after second injection. Scale bars, 100 μm. (D) Quantitative violin plots showing mean fluorescence intensity (MFI) of NAMPT within specific cell marker-labeled areas (GFAP for astrocytes and IBA1 for microglia) between PBS and LPS groups. (Cell numbers: GFAP [PBS = 95, LPS = 225], IBA1 [PBS = 116, LPS = 187]). ∗∗∗∗*p* < 0.0001 (Student’s *t* test). (E and F) Primary cortical astrocytes (DIV10) (E) and BV2 (F) were treated with 1 μg/mL LPS for 24 h and the resulting CMs were immunodepleted using control IgG or anti-NAMPT antibody. HT22 cells expressing the C99-GVP luciferase reporter were then incubated with control CM, NAMPT-depleted CM, or NAMPT-depleted CM supplemented with 1 μg/mL rNAMPT for 24 h, and γ-secretase activity was assessed using a luciferase reporter assay. (G) HT22 cells were co-transfected with C99-GVP, UAS-Luc, and LacZ for 24 h, treated with 1 μg/mL rNAMPT protein or heat-denatured rNAMPT protein for additional 24 h in the absence or presence of polymyxin B pre-incubation. Then, γ-secretases activity was assessed using a C99-GVP luciferase reporter assay. (H and I) rNAMPT does not affect Notch cleavage. HEK293T cells were transfected with NotchΔE-GFP for 24 h, treated with 1 μg/mL rNAMPT for an additional 24 h, and analyzed with western blotting (H). The signals of NICD on the blots were quantified using ImageJ (I). n.s., not significant. Bars represent mean ± SEM (E and F, *n* = 4 and 5; G, *n* = 4; I, *n* = 3) ∗*p* < 0.05, ∗∗*p* < 0.01, ∗∗∗*p* < 0.005, ∗∗∗∗*p* < 0.0001 n.s.; not significant (E, F; one-way ANOVA, G; two-way ANOVA, I; Student’s *t* test).

NAMPT is ubiquitously expressed in a variety of tissues, including adipose tissue, liver, and brain.^42^^,^^43^ To assess cell-type-specific NAMPT expression in the brain during neuroinflammation, we utilized a public single-cell RNA sequencing (scRNA-seq) dataset (GEO: GSE263094) from an LPS-induced mouse.^44^ Sub-clustering analysis yielded well-defined annotations of distinct cell lineages based on selective cell type markers (Figures S2A and S2B). The scRNA-seq profile revealed that *Nampt* transcript is ubiquitously distributed throughout brain cell types, with significantly upregulated within glial lineages following LPS treatment (Figures 2A and 2B). Notably, a highly reactive astrocyte subcluster characterized by elevated *Gfap* exhibited a significant enrichment of *Nampt* transcription (Figure 2A, enlarged). To validate the transcriptomic results *in vivo*, we performed immunofluorescence staining on brain sections from PBS- and LPS-treated mice (0.5 mg/kg/day for 2 days, intraperitoneal [i.p.]) using cell-type-specific markers. Consistently, LPS treatments showed significantly elevated NAMPT levels in astrocytes, alongside a shift toward a high-expressing population in microglia (Figures 2C and 2D). In contrast, neurons showed only a marginal increase in NAMPT intensity, while endothelial cells exhibited no significant change of NAMPT intensity (Figure S2C). Furthermore, we validated that NAMPT was highly expressed in primary cortical astrocytes, moderate in primary cortical microglia, and relatively low in primary cortical neurons (Figure S2D).

We then addressed whether glia-derived eNAMPT regulated γ-secretase activity in neuronal cells. When HT22 hippocampal cells were incubated with conditioned media prepared from either primary cortical astrocytes or BV2 mouse microglial cells which were exposed to LPS, γ-secretase activity was significantly increased (Figures 2E and 2F). Crucially, immunodepleting the CM with anti-NAMPT antibody blocked the stimulation of γ-secretase activity. Again, supplementation of NAMPT-depleted CM with excess recombinant NAMPT protein (rNAMPT) re-elevated γ-secretase activity (Figures 2E and 2F). Intriguingly, treatment with rNAMPT alone significantly increased γ-secretase activity, whereas heat-denatured rNAMPT failed to do so. In addition, polymyxin B, an LPS-neutralizing reagent, did not block the stimulatory effect of rNAMPT on γ-secretase activity either, indicating that the observed response is driven by its intrinsic protein structure (Figure 2G). In contrast, this stimulatory effect of rNAMPT was not observed in Notch cleavage (Figures 2H and 2I). Together, these results indicate that glia-derived eNAMPT stimulates γ-secretase activity in neuronal cells.

### The rNAMPT directly binds to C-terminal domain of IFITM3 on cell surface

To assess how eNAMPT regulates γ-secretase in neuronal cells, rNAMPT protein was purified from *E. coli* and fluorescently labeled with DyLight 488 NHS ester (rNAMPT-488) (Figure S3A). Upon treatment with rNAMPT-488 for 30 min or 1 h, the fluorescence was initially detected at the wheat germ agglutinin (WGA)-positive cell surface and internalized within Rab7-positive endocytic vesicles in HT22 cells (Figures S3B and S3C). Pretreatment with endocytosis inhibitor, methyl-β-cyclodextrin (MβCD) did not much affect the initial cellular binding of eNAMPT, whereas preincubation with anti-NAMPT antibody effectively blocked it (Figures S3D and S3E). A similar pattern of cellular binding of rNAMPT was also observed in mouse embryo fibroblasts (MEFs) (Figures S3F and S3G). We then hypothesized that there might be a binding partner for eNAMPT on cell surface, which functions to modulate γ-secretase activity. Under inflammatory conditions, IFITM3 has emerged as a strong candidate, as it is localized at the plasma membrane and endolysosomal compartments^45^^,^^46^^,^^47^ and is associated with presenilin in the γ-secretase complexes.^34^ Previously, a yeast two-hybrid screening also showed a possible interaction between IFITM3 and NAMPT.^48^

**Figure 3:**
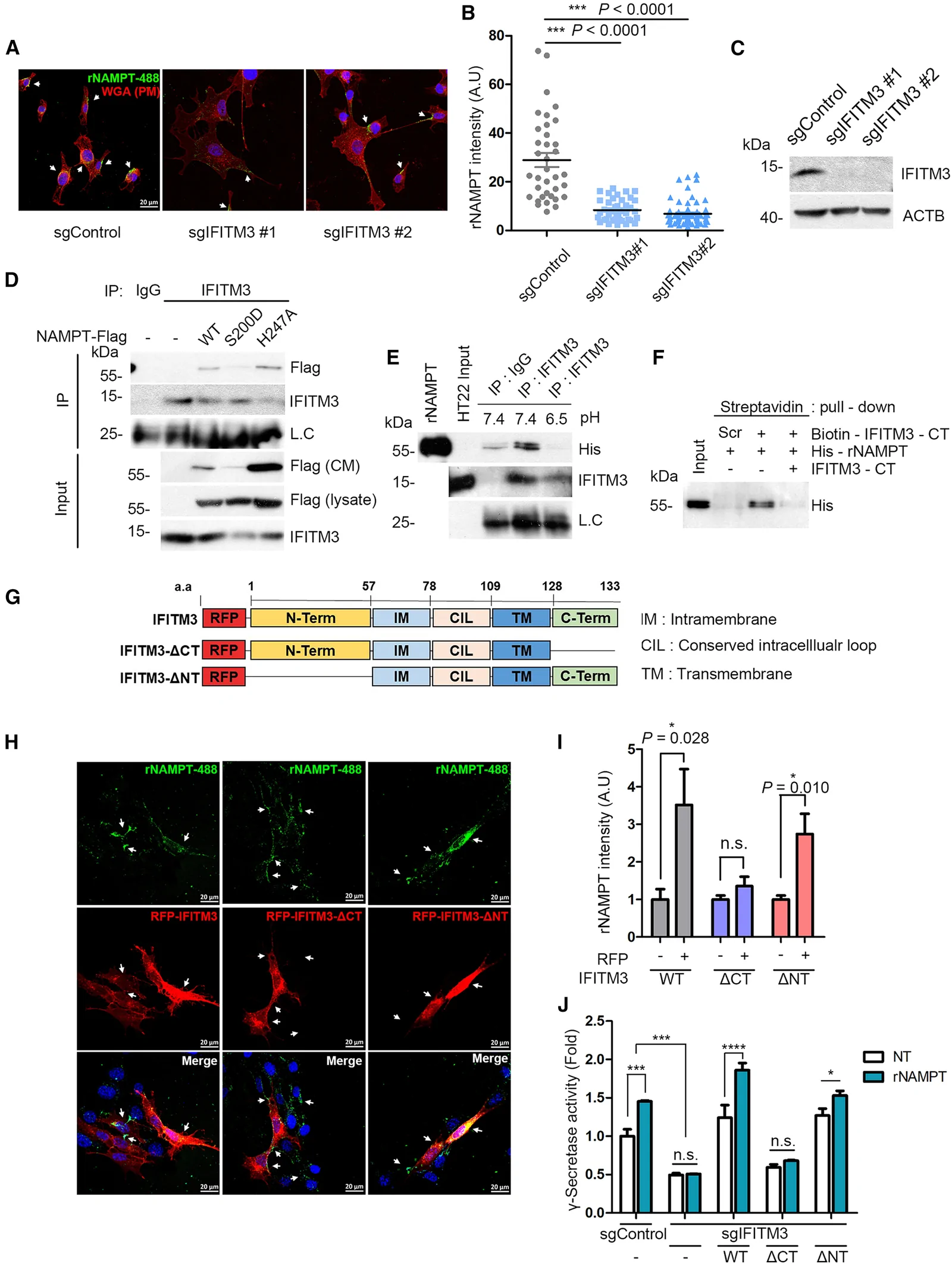
The C-terminal domain of IFITM3 is essential for rNAMPT-binding and subsequent γ-secretase stimulation (A and B) HT22 sgControl and sgIFITM3 cells were treated with 1 μg/mL rNAMPT-488 for 1 h, stained as described the aforementioned, and imaged using LSM700 confocal microscope. Scale bars, 20 μm (A). Cellular signals of rNAMPT-488 were quantified using ImageJ. Bars represent mean ± SEM (number of cells per group: 36, 41, and 71, ∗∗∗*p* < 0.005, Student’s *t* test) (B). (C) HT22 sgControl and sgIFITM3 cell lysates were analyzed by western blotting. (D) HEK293T cells were transfected with FLAG-tagged NAMPT WT, H247A dominant-negative, or S200D monomeric mutant for 24 h and then subjected to immunoprecipitation (IP) assay using anti-IFITM3 or control IgG antibody. Input, 10%. (E) Crude membrane fractions of HT22 cells were solubilized with 0.5% n-Dodecyl beta-D-maltoside (DDM) for 1 h and incubated with buffer (different pH) containing 0.5 μg rNAMPT for 1 h at 37°C. Samples were then subjected to immunoprecipitation (IP) assay using anti-IFITM3 or control IgG antibody. Input, 10%. (F) Streptavidin beads immobilized with 1 nM biotinylated IFITM3-CT peptide (biotin-IFITM3-CT) or biotinylated scrambled peptide (Scr) were incubated with 1 μg purified rNAMPT protein for 2 h. For the competition assay, rNAMPT was pre-incubated with excess IFITM3 CT peptide (IFITM3-CT) before the addition to IFITM3 CT peptide-immobilized beads. Bound rNAMPT was detected by western blotting. (G) Domain structure of IFITM3 WT and deletion mutants (Δ). N-term, N-terminus; IM, intramembrane; CIL, conserved intracellular loop; TM, transmembrane; C-term, C-terminus. (H and I) HT22 cells were transfected with IFITM3 WT-RFP or deletion mutant-RFP for 48 h, and then kept on ice, followed by the treatment with 1 μg/mL rNAMPT-488 for 10 min. Cells were stained and observed as described in Figure 3A. Arrows indicate localization of rNAMPT-488. Scale bars, 20 μm. Cellular signals of rNAMPT-488 were quantified using ImageJ. Bars represent mean ± SEM (number of cells per group RFP-IFITM3; RFP−/+ = 10, 10 RFP-IFITM3 ΔCT; RFP−/+ = 15, 12 RFP-IFITM3 ▵NT; RFP−/+ = 14, 10) ∗*p* < 0.05, n.s., not significant (Student’s *t* test) (I). (J) SH-SY5Y sgControl and sgIFITM3 cells were co-transfected with C99-GVP luciferase reporter system (C99-GVP, UAS-Luc, and LacZ) along with ITITM3-WT, -ΔCT or -ΔNT mutant for 24 h, treated with/without 1 μg/mL rNAMPT for additional 24 h, and assessed for γ-secretases activity using a C99-GVP luciferase reporter assay. Bars represent mean ± SEM (*n* = 4), ∗*p* < 0.05, ∗∗*p* < 0.01, ∗∗∗*p* < 0.005, ∗∗∗∗*p* < 0.0001 n.s., not significant (two-way ANOVA).

We thus examined the binding of eNAMPT to IFITM3 on the cell surface. Intriguingly, cellular binding of rNAMPT-488 was markedly reduced in IFITM3 knockout (KO) HT22 cells compared to control cells (Figures 3A–3C). Using co-immunoprecipitation (coIP) assays, we also found that IFITM3 bound to NAMPT WT and H247A mutant, but not to secretion-defective NAMPT S200D mutant (Figure 3D). Furthermore, the results of *in vitro* binding assays revealed that rNAMPT bound to IFITM3 at neutral pH 7.4, with the binding affinity markedly reduced at pH 6.5 (mimicking the pH of endosomal vesicles) (Figure 3E). To assess whether rNAMPT directly binds to IFITM3, we performed *in vitro* binding assays between purified rNAMPT protein and a biotinylated C-terminal peptide of IFITM (biotin-IFITM3-CT). Streptavidin pull-down of biotin-IFITM3-CT successfully co-precipitated rNAMPT, which was inhibited by an excess of cold C-terminal peptide of IFITM3 (IFITM3-CT) (Figure 3F). Together, these results imply that rNAMPT directly binds to IFITM3 on cell surface through its C-terminus domain and this interaction might be weakened upon internalization into acidic endosomal compartments, allowing the detachment of NAMPT from IFITM3.

We then examined the IFITM3 region involved in the rNAMPT-binding using IFITM3 deletion mutants lacking the C-terminal extracellular domain (ΔCT), or N-terminal intracellular domain (ΔNT) (Figure 3G). Expression levels of RFP-fused IFITM3 WT and its deletion mutants were similar in the transfected HT22 cells. Following incubation with rNAMPT-488, the cellular signal of rNAMPT-488 was highly observed in HT22 cells expressing RFP-IFITM3 or RFP-IFITM3-ΔNT, while it was low in cells expressing IFITM3-ΔCT compared to that in untransfected adjacent cells (Figures 3H and 3I). To validate whether the C-terminus of IFITM3 is functionally essential for γ-secretase activity, we performed a rescue experiment in SH-SY5Y IFITM3 KO cells. γ-Secretase activity increased by the reconstitution with IFITM3 WT and ΔNT, but not with IFITM3 ΔCT upon NAMPT treatment (Figure 3J). These results indicate that the C-terminal extracellular region of IFITM3 is required for cellular binding to rNAMPT and for mediating the stimulating activity of NAMPT for γ-secretase.

### NAMPT regulates IFTIM3-associated γ-secretase activity under inflammatory conditions

**Figure 4:**
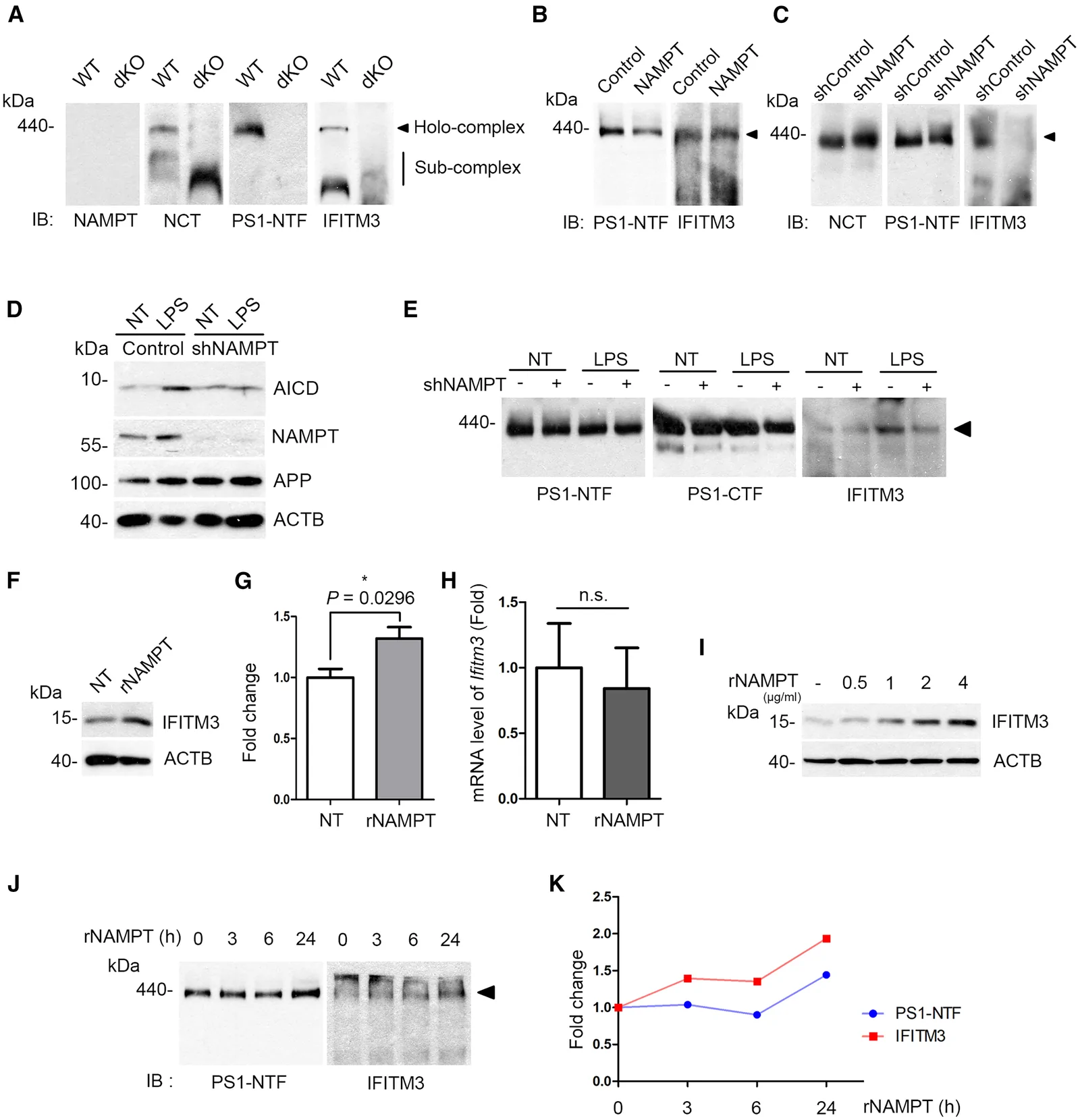
NAMPT modulates IFITM3-associated γ-secretase complexes (A) Crude membrane fractions prepared from MEF WT and PS1/2 double knockout (dKO) cells and solubilized in 0.5% DDM for 2 h were analyzed by BN-PAGE and western blotting. Arrowhead indicates γ-secretase holoenzyme complexes. (B and C) HEK293T (B) and SH-SY5Y/APPsw (C) cells were transfected with NAMPT-FLAG for 24 h (B) or shNAMPT for 48 h (C). The γ-secretase complexes in crude membrane fractions were analyzed by BN-PAGE followed by western blotting. Arrow heads indicate γ-secretase holoenzyme complexes. (D) SH-SY5Y/APPsl-PS1ΔE9 cells were transfected with control or shNAMPT for 24 h, followed by the treatment with 1 μg/mL LPS for an additional 24 h. Crude membrane fractions were subjected to an *in vitro* AICD generation assay for 2 h and analyzed by western blotting. (E) SH-SY5Y/APPsl-PS1ΔE9 cells were transfected with control or shNAMPT for 24 h and treated with 1 μg/mL LPS for additional 24 h. Crude membrane fractions solubilized with 0.5% DDM for 2 h were analyzed by BN-PAGE and western blotting. Arrowhead indicates γ-secretase holoenzyme complexes. (F and G) HT22 cells were treated with 1 μg/mL rNAMPT for 1 h and analyzed by western blotting (F). IFITM3 levels on the blots were quantified using ImageJ (G). Bars represent the mean ± SEM (*n* = 5), ∗*p* < 0.05 (Student’s *t* test). (H) HT22 cells were treated with 1 μg/mL rNAMPT for 1 h. Total RNAs were isolated from the harvested HT22 cells by Trizol. The mRNA levels of Ifitm3 were analyzed by QuantStudio qPCR. Actb is used for reference genes. (I) HT22 cells were treated with the indicated concentrations of rNAMPT for 1 h and analyzed by western blot analysis. (J and K) HT22 cells were incubated with 1 μg/mL rNAMPT for the indicated times. Crude membrane fractions solubilized with 0.5% DDM for 2 h were analyzed by BN-PAGE and western blotting. Arrowhead indicates γ-secretase holoenzyme complexes. PS1-NTF and IFITM3 levels on the blots were quantified using ImageJ.

The notion that eNAMPT binds to IFITM3 led us to examine its potential role in γ-secretase complex formation. We first analyzed the size and composition of the γ-secretase holoenzyme complex. Using blue native (BN)-PAGE analysis, we detected an IFITM3-associated γ-secretase holo-complex (NCT and PS1-NTF) with approximately 440 kDa, which corresponds to the size of γ-secretase holoenzymes in WT MEFs, but not in PS1/2 double KO (dKO) MEFs (Figure 4A). We confirmed that NAMPT overexpression did not alter γ-secretase holoenzyme levels, but it increased IFITM3 levels incorporated into γ-secretase complexes (Figure 4B). Intriguingly, the association of IFITM3 with γ-secretase complexes was abolished by NAMPT knockdown in SH-SY5Y/APPsw cells, despite total levels of γ-secretase complexes remaining unchanged (Figure 4C). IFITM3 knockdown suppressed AICD generation, reflecting the reduced γ-secretase activity, without affecting the levels of γ-secretase complexes (Figures S4A–S4C). We also confirmed that NAMPT overexpression and knockdown did not alter the levels of IFITM3 and γ-secretase components, such as NCT, PS1-NTF, APH1a, and PEN2 (Figures S4D–S4G). Together, these findings suggest that NAMPT modulates formation of the IFITM3-associated γ-secretase complex.

We then explored the role of the eNAMPT-IFITM3 axis under inflammatory conditions. Treatment of SH-SY5Y/APPsl-PS1ΔE9 cells with LPS increased AICD and NAMPT levels (Figure 4D). However, this increase in AICD by LPS was abolished by NAMPT knockdown in the same cells. In parallel, BN-PAGE analysis revealed that LPS treatment increased levels of the IFITM3-associated γ-secretase complexes, but not in NAMPT knockdown cells (Figure 4E). Thus, NAMPT enhances formation of the IFITM3-associated γ-secretase complexes under inflammatory conditions. In addition, treatment of HT22 cells with rNAMPT increased IFITM3 protein levels in a dose-dependent manner as early as 1 h (Figures 4F, 4G, and 4I), without changing IFITM3 mRNA levels (Figure 4H). This increase in IFITM3 protein was still observed in the presence of C34, a TLR4-selective inhibitor^49^ (Figure S5A). Furthermore, rNAMPT treatment delayed the degradation of IFITM3 protein in the presence of protein synthesis inhibitor, cycloheximide (Figure S5B). Given that IFITM3 is degraded by the ubiquitin-proteasome system (UPS),^50^ we also validated that rNAMPT treatment affected IFITM3 protein stability by reducing its ubiquitination (Figures S5C and S5D). Consistently, rNAMPT treatment increased levels of the IFITM3-associated γ-secretase complex after 3 h (Figures 4J and 4K). These results indicate that NAMPT increases IFITM3 protein levels primarily via enhancing protein stability.

### NAMPT levels are elevated in the CSF and cortex of AD patients and AD model mice

**Figure 5:**
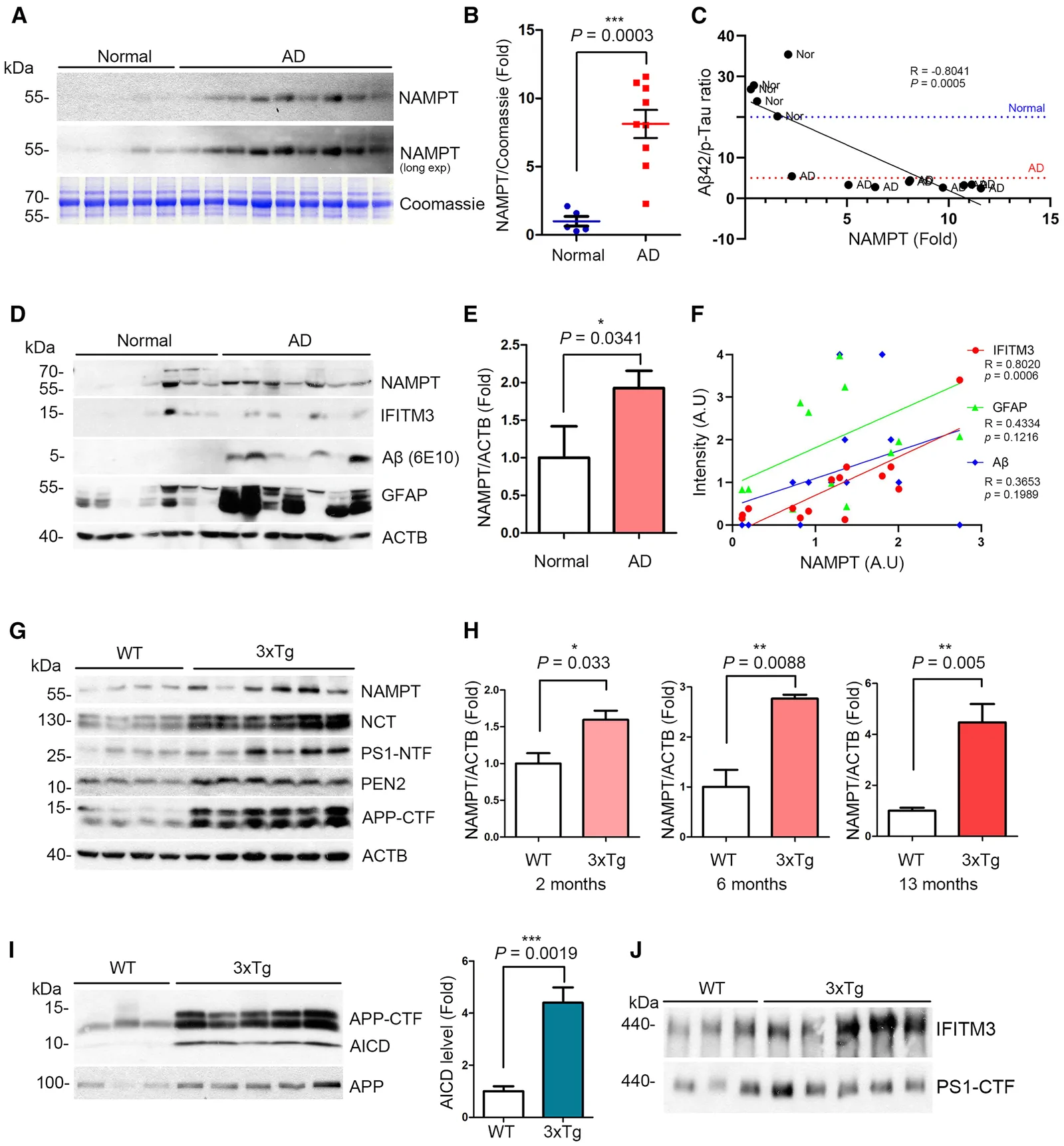
NAMPT is elevated in the CSF and cortex of AD patients and 3xTG mice (A and B) CSF samples of patients with AD and non-AD were analyzed by western blotting. Coomassie blue staining was used as a loading control (A). Relative levels of NAMPT on the blots to Coomassie blue staining were measured by ImageJ (B). (C) Linear correlation analysis between NAMPT and Aβ42/p-Tau ratio in CSF samples of patients with AD and non-AD in (A). (D and E) Levels of NAMPT and IFITM3 in the cortex of patients with AD and non-AD were assessed by western blotting (D). Relative levels of NAMPT to tubulin on the blots were determined by ImageJ. One sample was excluded based on Grubbs’ outlier test (E). (F) Linear correlation analysis between NAMPT and IFITM3 (red), GFAP (green), or Aβ levels (blue) in the cortex of patients with AD and non-AD in (D). (G and H) Levels of NAMPT, APP, and γ-secretase components in the cortex of 3xTg mice (13-month) were assessed by western blotting (G). The signals of NAMPT on the blots were quantified using ImageJ (H). (*n*; 2-month-old WT = 3, 2-month-old 3xTg = 3, 6-month-old WT = 5, 6-month 3xTg = 3, 13-month-old WT = 4, 13-month-old 3xTg = 6). (I) Crude membrane fractions of WT and 3xTg (13-month) cortex were subjected to *in vitro* AICD generation assay, followed by western blotting (left). The signals of AICD on the blots were quantified using ImageJ. n.s., not significant (right). (J) Crude membrane fractions from WT and 3xTg (13-month) cortex solubilized with 0.5% DDM were analyzed with BN-PAGE and western blotting. Values and bars represent mean ± SEM ∗*p* < 0.05, ∗∗*p* < 0.01, ∗∗∗*p* < 0.005 (Student’s *t* test) (B, E, H, and I).

To address the pathologic relevance of NAMPT in AD, we analyzed its levels in the cerebrospinal fluid (CSF) and mid-cortex brain tissue prepared from AD patients. Western blot analysis demonstrated a significant and drastic increase of NAMPT levels in the CSF samples of AD patients compared to non-AD CSF samples, with an average fold change of approximately 8.3 (Figures 5A and 5B). In addition, we analyzed established AD biomarkers and compared with NAMPT. AD and non-AD groups were separated by CSF Aβ42 and phosphorylated Tau (p-Tau181) levels (Figure S6A). Correlation analysis revealed that NAMPT levels showed a negative correlation with CSF Aβ42 (R = −0.8472) and a positive correlation with p-Tau181 (R = 0.6394), but no significant association with total Tau (R = 0.4841) (Figure S6B). Given that Aβ42/p-Tau ratio is widely utilized as a diagnostic index for AD, we also examined its correlation with NAMPT levels and found a strong inverse correlation between CSF NAMPT and the Aβ42/p-Tau ratio (R = −0.8041). Thus, high NAMPT levels are associated with a more pronounced AD biomarker profile (Figure 5C).

We also found a significant increase of NAMPT protein in the mid-cortex brain tissue of AD patients, showing a 1.8-fold increase compared to non-AD controls (Figures 5D and 5E). Unlike proteins, however, analysis of NAMPT mRNA levels using multiple datasets (GSE36980 and GSE5281) revealed no significant changes in the hippocampus, temporal cortex, frontal cortex, and entorhinal cortex between AD patients and non-AD patients (Figures S6C–S6F). In addition, Pearson correlation analysis of protein levels in AD mid-cortex revealed R = 0.80, a strong positive relationship between NAMPT and IFITM3 (Figures 5D and 5F). We also found a statistically insignificant but positive correlation between NAMPT and astrocyte activation marker GFAP, (R = 0.43) or Aβ, (R = 0.37) (Figures 5D and 5F). These results imply that the increase of NAMPT levels in AD brain tissues and CSF may be associated with neuroinflammation and amyloidogenic processes.

We then analyzed NAMPT levels in 3xTg-AD mice, a widely used AD model expressing APPswe, PS1M146V, and Tau P301L.^51^ Compared to age-matched WT controls, levels of NAMPT protein in 3xTg mice were significantly elevated at 2, 6, and 13 months of age (Figures 5G and 5H). The increase was age-dependent, with 1.6-fold at 2 months, 2.7-fold at 6 months, and 4.4-fold at 13 months. On the other hand, there were no significant changes in NAMPT mRNA levels (Figure S7A). As seen in the increase of NAMPT levels, we also observed an age-dependent increase of AICD levels in the 3xTg-AD mouse brain (Figures 5I, S7B, and S7C), along with an increase in the IFITM3-associated γ-secretase complexes in the brains of 6-month-old and older mice (Figures 5J, S7D, and S7E). Interestingly, we observed an increase of γ-secretase components, including NCT, PS1-NTF, PEN2, and APP-CTF, particularly in the cortex of 6- and 13-month-old 3xTg mice, but not in 2 months of age (Figures 5G and S7F). Similar patterns were also observed in IL-6 mRNA levels (Figure S7G). Thus, there is a considerable association between NAMPT levels and AD pathogenesis in 3xTg mice.

### LPS administration promotes NAMPT-IFITM3 association and γ-secretase activation in WT and APP-NL-G-F mice

**Figure 6:**
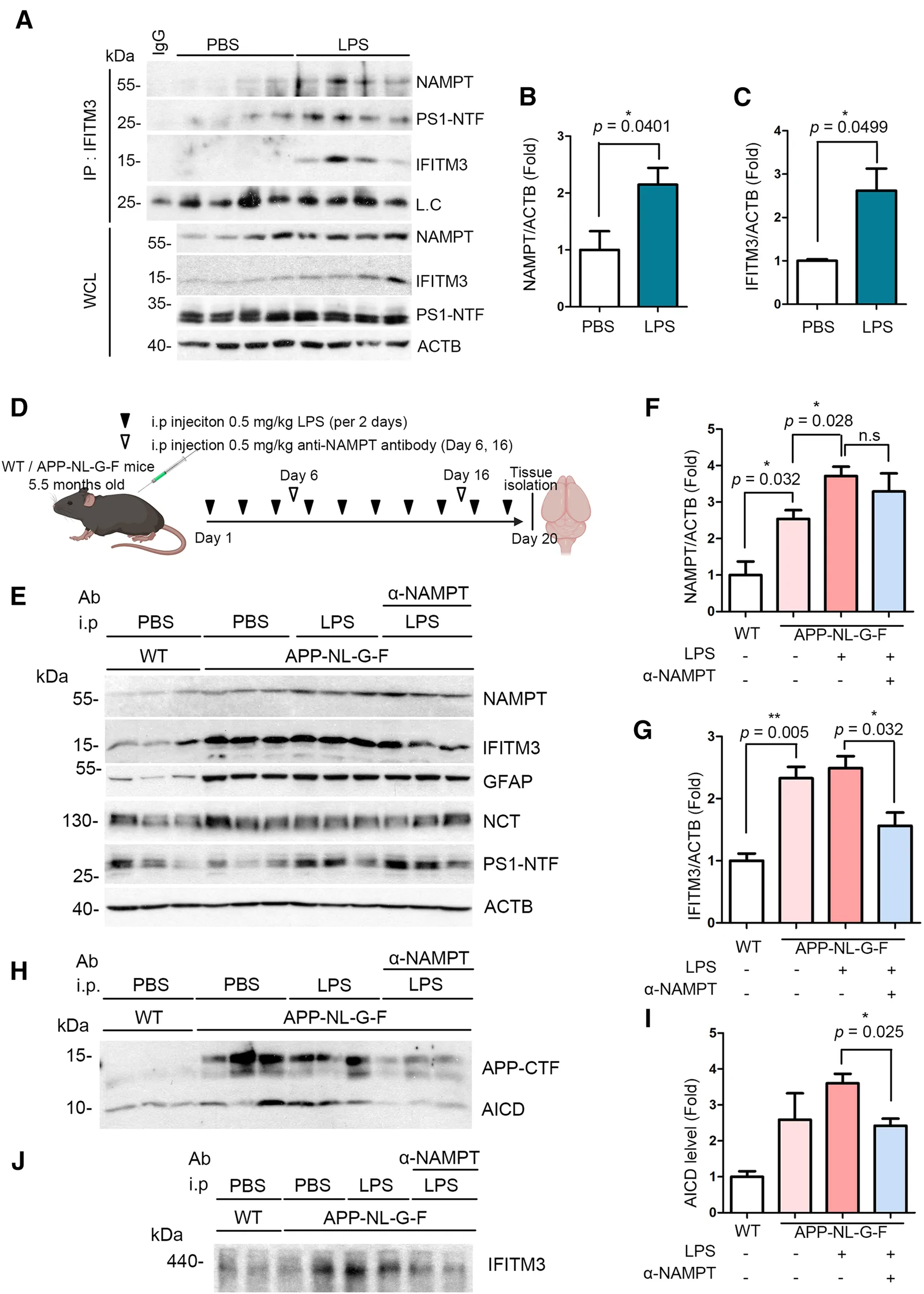
LPS-induced inflammation promotes NAMPT-IFITM3 association and IFITM3-associated γ-secretase levels in WT and APP-NL-G-F mice (A–C) 3-month-old C57BL/6 wild-type mice were intraperitoneally and daily injected with 0.25 mg/kg LPS or PBS for 7 days. Hippocampal tissues were analyzed by western blotting. Crude membrane fractions of the hippocampus solubilized in 0.5% DDM were subjected to the immunoprecipitation (IP) assay using an anti-IFITM3 antibody or control IgG (A). The signals of NAMPT (B) and IFITM3 (C) on the blots in (A) were quantified using ImageJ. Bars represent mean ± SEM (*n*; PBS = 4, LPS = 4), (Student’s *t* test). (D) Scheme showing intraperitoneal (i.p.) injection of LPS and anti-NAMPT antibody into APP-NL-G-F mice. (E–G) 5.5-month-old C57BL/6 WT and APP-NL-G-F mice were intraperitoneally injected with 0.5 mg/kg LPS or PBS every 2 days for a total of 10 injections over 20 days, and with anti-NAMPT antibody (0.5 mg/kg) on days 6 and 16. Cortical tissues were analyzed by western blotting (E) and the signals of NAMPT (F) and IFITM3 (G) on the blots were quantified using ImageJ. (H–J) Crude membrane fractions prepared from the cortex of the same mice were subjected to *in vitro* AICD generation assay, followed by western blotting (H). The signals of AICD on the blots were quantified using ImageJ (I). Crude membrane fractions solubilized with 0.5% DDM were analyzed with BN-PAGE and western blotting (J). Bars represent mean ± SEM (B, C, F, G, and I). ∗*p* < 0.05, ∗∗*p* < 0.01, n.s., not significant; (Student’s *t* test).

To examine whether inflammatory stimulation engages the NAMPT–IFITM3 axis *in vivo*, we first utilized 3-month-old C57BL/6 WT mice. Daily i.p. injection with 0.25 mg/kg LPS for 7 days, which induces systemic inflammation to induce neuroinflammatory responses,^52^^,^^53^ significantly increased NAMPT and IFITM3 levels in the hippocampal tissues compared to controls (Figures 6A–6C). Intriguingly, coIP analysis of the same hippocampal tissue extracts revealed that the association between NAMPT and IFITM3 was enhanced in the LPS-treated group compared to controls, as well as those between IFITM3 and presenilin 1 (Figure 6A).

We then investigated this mechanism in a relevant AD mouse model. We utilized 5.5-month-old APP-NL-G-F knock-in mice, an AD model expressing humanized APP with Swedish (KM670/671NL), Arctic (E693G), and Iberian (I716F) mutants at endogenous levels.^54^^,^^55^ Mice were intraperitoneally injected with 0.5 mg/kg LPS every two days for a total of 10 injections over 20 days. Anti-NAMPT antibody was administered intraperitoneally on day 6 and 16 to deplete eNAMPT (Figure 6D). Interestingly, NAMPT levels were significantly higher in the cortex of APP-NL-G-F mice than in age-matched WT mice and further increased upon LPS treatment (Figures 6E and 6F). While NAMPT levels were not affected by anti-NAMPT antibody injections, IFITM3 levels were dramatically reduced (Figures 6E and 6G). We also found that AICD levels in the cortical tissue were significantly suppressed by anti-NAMPT antibody treatment (Figures 6H and 6I). Furthermore, BN-PAGE analysis revealed that the IFITM3-associated γ-secretase levels were reduced by anti-NAMPT antibody (Figure 6J). Collectively, these findings indicate that the inflammation-driven increase of NAMPT promotes the IFITM3 incorporation into γ-secretase components and enhances γ-secretase activity under AD-relevant pathological conditions.

## Discussion

In this study, we identify a non-canonical role of NAMPT in regulating γ-secretase activity, providing a crucial molecular link between neuroinflammation and AD pathogenesis. While NAMPT is a multifunctional protein primarily known for its role in NAD+ biosynthesis, eNAMPT, a cytokine-like molecule in metabolic and inflammatory diseases, modulates γ-secretase without affecting α- or β-secretases. In this regulation, the enzymatic activity of NAMPT is dispensable. The elevation of NAMPT levels in the CSF and cortical tissues of AD patients and in 3xTg and APP-NL-G-F AD mouse models provides its pathological relevance. As a non-canonical role, eNAMPT interacts with IFITM3, a known presenilin regulator, on cell surface, leading to increase of IFITM3 protein stability and enhanced formation of the IFITM3-associated γ-secretase complex. Previously, IFITM3 was shown to localize in lipid raft of the plasma membrane and mediate endocytic trafficking, an event linked to enhanced APP processing.^56^^,^^57^ Thus, eNAMPT largely derived from reactive glial cells acts as an upstream paracrine signal that stimulates endocytic vesicle trafficking through IFITM3 and amplifies γ-secretase activity in neurons.

The mechanism by which eNAMPT enhances the incorporation of IFITM3 into γ-secretase complex appears independent of the previously reported receptors. Although TLR4 and CCR5 were reported as eNAMPT receptors, several lines of evidence exclude their involvement in IFITM3 regulation.^24^^,^^58^^,^^59^^,^^60^^,^^61^ Specifically, the rapid increase of IFITM3 protein levels by rNAMPT treatment in the absence of changes in IFITM3 mRNA levels suggests that enhances post transcriptionally protein stability. Confirmatively, treatment with selective TLR4 inhibitor C34 also did not block eNAMPT-mediated increase of IFITM3 and CCR5 overexpression did not alter levels of γ-secretase activity and complex (data not shown). However, we cannot entirely exclude an alternative, unidentified receptor or signaling which modifies IFITM3 protein and regulates its protein stability.^62^^,^^63^

While previous studies have reported a decline in tissue NAMPT levels and its enzymatic activity during aging and pathological progression,^64^^,^^65^ our observation showing elevation of total NAMPT in AD brains could be comprehensively reconciled with those findings through several considerations. In pathological conditions, a preferential increase of NAMPT in monomeric form would lead to an increase in total protein abundance without a corresponding rise in enzymatic activity.^66^^,^^67^ In addition, neuronal NAMPT loss can also be counterbalanced or masked by the upregulation of NAMPT in reactive glial cells, such as astrocytes and microglia.^68^ Furthermore, methodological differences to measure membrane proteins may also affect the results; our studies represent levels of membrane-associated populations.

The functional role of NAMPT-IFITM3 axis under inflammation was further corroborated by *in vivo* experiments using a systemic LPS injection model, which induces neuroinflammation through peripheral immune activation.^52^^,^^53^ In the LPS-injected mice, we observed a marked increase of NAMPT levels in the brain. Accordingly, both NAMPT-IFITM3 and IFITM3-presenilin interactions also increased. However, it is interesting to note that distinct patterns of NAMPT induction in the brain emerged during acute and chronic inflammation paradigms. Under acute inflammatory conditions, increase of NAMPT mRNA levels in the brain appears to be primarily driven by glial cell activation (Figures S8A and S8B). On the other hand, NAMPT mRNA levels in the brain are not much affected under chronic inflammatory conditions, such as aging or sustained systemic inflammation. Thus, eNAMPT may also originate from the peripheral tissues, such as adipose tissue,^30^^,^^69^ and subsequently contribute to neuroinflammation cross blood-brain barrier. This highlights a crucial pathologic link between chronic metabolic disorders, such as type 2 diabetes, an AD risk factor, and AD pathology.

In summary, discovery of the eNAMPT-IFITM3 axis as a regulator of γ-secretase offers a highly promising therapeutic target for reducing amyloid pathology while preserving essential Notch signaling. The existing clinical interest in eNAMPT-neutralizing antibodies for inflammatory disease further provides therapeutic potential targeting eNAMPT to mitigate its role in γ-secretase regulation.^70^^,^^71^^,^^72^^,^^73^ Despite these advances, the precise molecular understanding how the eNAMPT-IFITM3 interactions influence γ-secretase remains to be further investigated.

### Limitations of the study

This study highlights the non-canonical role of NAMPT as a γ-secretase regulator and its potential relevance to AD pathology. However, there are notable limitations that require further investigation.

First, while a physical interaction between eNAMPT and IFITM3 was demonstrated, the precise mechanism by which this interaction influences γ-secretase activity remains unclear. Whether the IFITM3-associated γ-secretase complex dynamics change, the molecular mechanism how eNAMPT affects formation of IFITM3-associated γ-secretase complex is not fully understood. Although the interaction between eNAMPT and IFITM3 appears to influence the formation of IFITM3-associated γ-secretase complex, eNAMPT is not detectable within the final stable complex. This raises a possibility that eNAMPT may exert a transient influence on IFITM3 to enhance protein stability. Therefore, further structural and biochemical studies are needed to resolve these interaction dynamics.

Second, regarding *in vivo* validation and tissue-specificity, another limitation lies in confirming the role of eNAMPT in AD pathology. While LPS increases NAMPT levels and γ-secretase activity in mouse models, direct evidence linking NAMPT levels to amyloid deposition or cognitive decline is lacking. Studies employing knockdown or KO of NAMPT expression in AD mouse models are needed to confirm its pathological relevance. Furthermore, a significant challenge is to dissect the contributions of CNS or peripheral eNAMPT pools. In addition, the role of intracellular NAMPT could not completely be excluded. Previous studies have shown that only 1% of intracellular NAMPT is secreted into the extracellular space under normal conditions.^74^

Finally, it would be interesting to test whether circulating NAMPT originating from peripheral organs, such as adipose tissue, could also contribute to γ-secretase activation after crossing the blood-brain barrier. Therefore, future studies should adopt a more systemic and compartment-specific approach to dissect organ-specific and intra/extra roles of NAMPT, alongside further molecular characterization of its functional modes of action.

## Resource availability

### Lead contact

Further information and requests for resources should be directed to the lead contact, Yong-Keun Jung (ykjung@snu.ac.kr).

### Materials availability

This study did not generate new reagents.

### Data and code availability

• The scRNA-seq and transcriptomic datasets analyzed in this study are publicly available in the GEO database under accession number GSE263094 and are listed in the key resources table.
• This paper does not report original code.
• Any additional information required to reanalyze the data reported in this paper is available from the lead contact upon request.

## Acknowledgments

The authors would like to thank Dr. T Tomita (10.13039/501100004721University of Tokyo, Japan) for providing anti-PEN2 antibody. The authors would like to thank Prof. SY Kim (10.13039/100016275Seoul National University Bundang Hospital) and Prof. S-H Park (10.13039/100017051Seoul National University College of Medicine) for providing CSF and AD tissue. The authors would like to thank Prof. C Saido for providing APP NL-G-F mice (10.13039/501100006264RIKEN, Japan). This work was supported by a CRI grant no.(RS-2025-00519823), the Bio-medical Technology Development Program (RS 2024-00439842), 10.13039/501100003710Korea Health Industry Development Institute (HR22C141102) funded by the 10.13039/100009647Ministry of Health and Welfare and Ministry of Science, and Global-Learning and Academic Research Institution for Master’s·PhD students and Postdocs (G-LAMP) Program of the 10.13039/501100003725National Research Foundation of Korea (NRF) grant no.(RS-2024-00445180) funded by the Ministry of Education.

## Author contributions

J.H., K.W.K., and Y.-K.J. conceived the study; J.H., S.J., and J.N. carried out the experiments; Y.N. analyzed the scRNA-seq data; Y.C. performed the immunofluorescence in brain. J.H. and Y.-K.J. designed the experiments; J.H. and Y.-K.J. wrote the manuscript with input from all other authors. All authors approved the manuscript.

## Declaration of interests

The authors declare no competing interests.

## Declaration of generative AI and AI-assisted technologies in the writing process

During the preparation of this work the authors used ChatGPT to improve English language. After using this tool, the authors reviewed and edited the content as needed and took full responsibility for the content of the publication.

## STAR★Methods

### Key resources table

**Table:** 

REAGENT or RESOURCE	SOURCE	IDENTIFIER
**Antibodies**		

Anti-GFP	Santacruz	Sc-8334
Anti-His	Cell signaling	2365
Anti-Flag M2	Sigma-Aldrich	F1804
Anti-Flag	Cell signaling	14793
Anti-NAMPT	Santacruz	Sc-393444
Anti-NAMPT	Bethyl lab	BLR058F
Anti-IFITM3	Abclonal	A13070
Anti-Nicastrin	Sigma-Aldrich	N1660
Anti-Presenilin 1	Santacruz	Sc-7860
Anti-Presenilin 1	Abclonal	A2187
Anti-Aβ	Abclonal	A26342
Anti-Aβ 6E10	Convance	SIG-39320
Anti-APP	Sigma-Aldrich	A8718
Anti-APP	Abclonal	A17911
Anti-APH1	University of Tokyo	Dr. T.Tomita
Anti-PEN2	University of Tokyo	Dr. T.Tomita
Anti-Notch	Novus	NB200-251
Anti-GFAP	Cell signaling	80788
Anti-IBA1	Wako	019-19741
Anti-NeuN	MilliporeSigma	MAB377
Anti-CD31	Novus	NB600-562ss
Anti-Rab7	Abclonal	A12308
Anti-Ubiquitin	Santacruz	Sc-8017
Anti-GAPDH	Santacruz	Sc-365062
Anti-Tublin	Sigma-Aldrich	T6074
Anti-Actin	Sigma-Aldrich	A1978
Anti-mouse IgG	Santacruz	Sc-2025
Anti-rabbit IgG	Cell signaling	2729

**Bacterial and virus strains**		

BL21-Rosetta	N/A	N/A
Lentivirus shNAMPT-human	This paper	N/A
Lentivirus shNAMPT-mouse	This paper	N/A

**Biological samples**		

Human CSF samples	Seoul National University Bundang hospital (SNUBH)	neuroksy@snu.ac.kr
Human brain samples	Seoul National University Hospital Brain Bank (SNUHBB)	shparknp@snu.ac.kr
Brain samples from wild type control and 3xTg mice	This paper	N/A
Brain samples from wild type control and APP NL-G-F mice	This paper	N/A

**Chemicals, peptides, and recombinant proteins**		

Anti-flag M2 affinity gel	Sigma-Aldrich	A2220
GFP trap	ChromoTek	gta
DyLight488	Invitrogen	A11001
Compound E	MedChemExpress	HY-14176
Cycloheximide	Sigma-Aldrich	239764
Lipopolysaccharides from Escherichia coli O55:B5	Thermofisher	L2880
Beta nicotinamide adenine dinucleotide, sodium salt	Sigma-Aldrich	N0632
FK866	Cayman Chemicals	13287
Wheat Germ Agglutinin (WGA) conjugate 488	Biotium	29022
Wheat Germ Agglutinin (WGA) conjugates 640	Biotium	29026
LY2886721	Selleckchem	S2156
n-Dodecyl beta-D-maltoside	Sigma-Aldrich	850520P
Doxycycline	Sigma-Aldrich	D3447
OligodT	GenDEPOT	N/A
M-MLV reversetranscriptase	Enzynomics	RT001S
M-MLVbuffer	Enzynomics	B201
SYBR green master mixture	Applied biosystems	4367659
Lipofector-pMAX Reagent	APTABIO	AB-LF-M100
PEI	Sigma-Aldrich	919012
C34	Tocris	5373
Saponin	Sigma-Aldrich	47036
ToxinCleanic Endotoxin Removal Kit	Bioneer	K-7800
C-terminal extracellular peptide of IFITM3 and scramble peptide control	Peptron, Korea	N/A

**Critical commercial assays**		

Luciferase assay system	Promega	E1500

**Deposited data**		

Single-cell RNA-seq dataset	Yoon et al.^44^	GEO: GSE263094

**Experimental models: Cell lines**		

HEK293T	ATCC	CRL-3216
Lenti-X 293T	Takara	632180
SH-SY5Y	ATCC	CRL-2266
SH-SY5Y/APPsw	Sungkyunkwan University, South Korea	jodg@skku.edu
SH-SY5Y/APPslPS1ΔE9	This paper	N/A
BV2	ATCC	CRL-2467

**Experimental models: Organisms/strains**		

Mouse: C57BL/6J	Jackson laboratory	000664
Mouse: B6;129 Psen1tm1MpmTg (APPSwe, tauP301L)1Lfa/Mmjax	Jackson laboratory	004807
Mouse: C57BL/6J APP NL-G-F	Riken, Japan	takaomi.saido@riken.jp

**Oligonucleotides**		

Real-time PCR mouse Ifitm3 forward: TCCTGCCTGGGCTTCATAG	This paper	N/A
Real-time PCR mouse Ifimt3 reverse: ACCAAGGTGCTGATGTTCAGGC	This paper	N/A
Real-time PCR mouse Il1β forward: TGGACCTTCCAGGATGAGGACA	This paper	N/A
Real-time PCR mouse Il1β reverse: GTTCATCTCGGAGCCTGTAGTG	This paper	N/A
Real-time PCR mouse Il6 forward: TACCACTTCACAAGTCGGAGGC	This paper	N/A
Real-time PCR mouse Il6 reverse: CTGCAAGTGCATCGTTGTT	This paper	N/A
Real-time PCR mouse Tnfα forward: GGTGCCTATGTCTCAGCCTCTT	This paper	N/A
Real-time PCR mouse Tnfα reverse: GCCATAGAACTGATGAGAGGGAG	This paper	N/A
Real-time PCR human NAMPT forward: AGGGTTACAAGTTGCCACC	This paper	N/A
Real-time PCR human NAMPT reverse: CTCCACCAGAACCGAAGGCAAT	This paper	N/A
Real-time PCR mouse Nampt forward: CTGTGGCGGGAATTGCTCTA	This paper	N/A
Real-time PCR mouse Nampt reverse: GTCTTTCCCCCAAGCCGTTA	This paper	N/A
NAMPT S200D forward: CTACAGAGGAGTCTCTGACCAAGAGACTGCTGGC	This paper	N/A
NAMPT S200D reverse: GCCAGCAGT CTCTTGGTCAGAGACTCCTCTGTAG	This paper	N/A
Mutagenesis NAMPT H247A forward: CAGAACACAGTACCATAAC	This paper	N/A
Mutagenesis NAMPT H247A reverse: GTTATGGTACTGTGTTCTGCTG	This paper	N/A
sgRNA mouse IFITM3 #1 forward: CACCGAATATGAGGTGGCTGAGATG	This paper	N/A
sgRNA mouse IFITM3 #1 reverse: AAACCATCTCAGCCACCTCATATTC	This paper	N/A
sgRNA mouse IFITM3 #2 forward: CACCGATATGAGGTGGCTGAGATGG	This paper	N/A
sgRNA mouse IFITM3 #2 reverse: AAACCCATCTCAGCCACCTCATATC	This paper	N/A

**Recombinant DNA**		

Lentiviral shRNA vector pGIPZ-human NAMPT-IRES-tGFP	Horizon	V2LHS_254334
Lentiviral shRNA vector pGIPZ-mouse Nampt-IRES-tGFP	Horizon	V3LMM_485506
pET28a-His mouse Nampt	Addgene	25630
pC99-GVP	Han et al.^10^	N/A
pUAS-Luciferase	Han et al.^10^	N/A
pβ-galactosidase	Han et al.^10^	N/A
pCMV-NAMPT-flag	This paper	N/A
pCMV-NAMPT S200D-flag	This paper	N/A
pCMV-NAMPT H247A-flag	This paper	N/A
pSEAP-APPa-muta	Han et al.^11^	N/A
NotchΔE-GFP	This paper	N/A
pGFP-IFITM3	This paper	N/A
pRFP-IFITM3	This paper	N/A
pRFP-IFITM3-ΔNT	This paper	N/A
pRFP-IFITM3-ΔCT	This paper	N/A

**Software and algorithms**		

ImageJ	National Institutes of Health	N/A
GraphPad Prism11	GraphPad	N/A

### Experimental model and study participant details

#### Animals

C57BL/6J and 3xTg mice were purchased from The Jackson Laboratory. APP NL-G-F mice were gifted from Takaomi C Saido (Riken, Japan). All mice were housed and maintained in specific pathogen free (SPF) facility under a 12-h light/12-h dark cycle. Age details for each experiment are indicated in the figure legends. All animal care and experimental procedures were performed in accordance with institutional guidelines and approved by the IACUC of Seoul National University (Approval No. SNU-251219). Both male and female mice were used, and no significant sex-dependent differences were observed.

#### Humans

CSF samples from non-Alzheimer’s disease control subjects and patients with Alzheimer’s disease related to both Figures 5 and S6 were supplied by Seoul National University Bundang hospital. Brain samples from non-Alzheimer’s disease control subjects and patients with Alzheimer’s disease related to both Figure 5 were supplied by Seoul National University Hospital Brain Bank (SNUHBB). The collection and use of all human specimens were approved by the IRB (Approval No. E1806/002-012 and E2403/002-031). The detailed information on control subjects and patients with Alzheimer’s disease is listed in Tables S1 and S2.

#### Cell lines

Human embryonic kidney (HEK293T) cells, human neuroblastoma (SH-SY5Y) cells, and mouse microglial (BV2) cells were cultured in Dulbecco’s modified Eagle’s medium (DMEM) supplemented with 10% fetal bovine serum (FBS) and 1% penicillin-streptomycin at 37°C in a humidified 5% CO2 incubator. The cell lines were maintained from laboratory stocks and were not authenticated or tested for mycoplasma contamination. DNA transfection was conducted by using Lipofector or PEI following the manufacturer’s instruction.

#### Mouse primary cell culture

Mouse primary cortex was harvested at embryonic day 16.5–17.5 and chunks of cortical neurons were incubated in Hanks’ Balanced Salt solution (HBSS) containing trypsin EDTA (TE, final 0.05%) for 20 min at 37°C. After inhibiting TE by adding fetal bovine serum (FBS, final 5%), neurons and astrocytes were isolated by following different culturing procedures. Neurons were dissociated with gentle pipetting and plated in culture dish under neurobasal medium supplemented with B27 supplement, Glutamax and Penicillin-Streptomycin at 37° under 5% CO2 (v/v). For astrocyte and microglia culture, postnatal day 0–1 cortical tissues were dissociated with gentle pipetting and cells were plated in T75 culture dish under Dulbecco’s modified Eagle’s medium (DMEM) supplemented with FBS (10%). After 7 to 8 days, cultured cells were incubated in 180 rpm shaker for 30 min to detach sheeted microglia. Detached microglia were centrifuged 180 *g* for min then plated in culture dish under DMEM supplemented with FBS (final 10%). To remove sheeted oligodendrocytes, T75 culture dish was further incubated in 240 rpm shaker for 6 h and then shaken vigorously for 1 min. Remaining Astrocytes were detached by treatment of TE and centrifuged 180 *g* for 5 min. Astrocytes were plated in culture dish under DMEM supplemented with FBS (final 10%). Over 14 days *in vitro* (DIV) astrocytes were used in our experiments.

#### Recombinant His-NAMPT protein

BL21-Rosetta bacterial strain was transformed with the pET28-His-NAMPT (Addgene plasmid #25630) vector. Transformed bacteria were grown in LB broth containing kanamycin (50 μg/mL) at 37°C until reached an optical density (OD_600_) of 0.5. Protein expression was induced by adding 1 mM isopropyl β-D-1-thiogalactopyranoside (IPTG), and the culture was further incubated at 18°C for 3 h to optimize protein folding. The bacterial culture was harvested by centrifugation, and the pellet was resuspended in lysis buffer (20 mM Tris-HCl, 500 mM NaCl, 10 mM imidazole, pH 8.0). To ensure efficient cell lysis, the suspension was sonicated on ice for 2 h (5-s pulse on, 5-s pulse off) at 30% amplitude. The lysate was clarified by centrifugation at 12,000 g for 30 min at 4°C to remove cellular debris. The supernatant containing the His-tagged NAMPT protein was incubated with Ni-NTA agarose beads (Qiagen) pre-equilibrated in lysis buffer overnight at 4°C with gentle rotation. The beads were washed five times with wash buffer (20 mM Tris-HCl, 500 mM NaCl, 10 mM imidazole, pH 8.0) to remove non-specifically bound proteins. His-NAMPT was eluted using elution buffer (20 mM Tris-HCl, 500 mM NaCl, 400 mM imidazole, pH 8.0). The purity of the eluted recombinant NAMPT protein was assessed by SDS-PAGE followed by Coomassie Brilliant Blue staining. Protein concentration was determined by comparison with a BSA standard curve. Endotoxin was removed using the ToxinCleanic Endotoxin Removal Kit (Bioneer) according to the manufacturer’s instructions.

#### Lentivirus generation

Lenti-X 293 T cell Line was used to generate lentivirus. The cells were co-transfected with PAX2, VSVG and either pGIPZ empty vector or pGIPZ-shNampt-IRES-tGFP for knockdown using pMAX reagent following manufacturer’s instruction. 60 h after transfection, harvested medium was filtered (40 μm pore) and incubated in lenti-X concentrator (Takara) for 30 min at 4°C. Finally, viral suspension was centrifuged at 1,500 g for 45 min and viral pellet was resuspended with PBS and stored at −70°C.

### Method details

#### Antibody application summary

Antibodies were selected based on application compatibility, assay sensitivity, and host-species requirements for co-immunostaining or immunoprecipitation–immunoblotting (IP–IB). For targets where multiple antibody clones or catalog numbers were utilized, specific antibodies were assigned to designated figure panels as follows:

• Anti-Flag antibody (Sigma, F1804): Used in Figures 1B, 1C, 1K, 3D, S1D, and S4D.
• Anti-Flag antibody (Cell signaling, 14793): Used in Figures 3D and S1D.
• Anti-NAMPT (Santacruz, 393444): Used in Figures 1E, 1F, 2C, 2E, 2F, 4D, 5A, 5D, S2C, S2D, S2E, S2G, and S4D.
• Anti-NAMPT (Bethyl lab, BLR058F): Used in Figures 5G, 6A, 6E, S7F, and S8A.
• Anti-Presenilin 1 (Santacruz, 7860): Used in Figures 4A–4C, 4E, 4J, 5G, 6A, 6E, S4B, S4D, S4F, and S7F.
• Anti-Presenilin 1 (Abclonal, A2187): Used in Figures 4E, 5J, S4B, S7D, and S7E.
• Anti-Aβ (Abclonal, A26342): Used in Figures 1C and 1F.
• Anti-Aβ (Convance, SIG-39320): Used in Figures 5D, S1A, and S8A.
• Anti-APP (Abclonal, A17911): Used specifically for AICD detection.

#### *In vitro* binding and competition assay

The C-terminal extracellular peptide of IFITM3 (LIFQAYG, IFITM3-CT) and a scrambled control (GQILAFY, Scr) were synthesized (Peptron, Korea) as free or N-terminally biotinylated peptides. Biotinylated IFITM3 CT peptide (Biotin-IFITM3-CT) and biotinylated scrambled peptide (Scr) were immobilized on streptavidin beads to examine their interaction with recombinant NAMPT. Beads were incubated with 1 nM of Biotin-IFITM3-CT peptide, or Scr in 100 μL binding buffer for 30 min at 4°C with gentle rotation. Unbound peptide was removed by washing the beads three times with binding buffer. For binding reactions, peptide immobilized beads were incubated with 1 μg purified rNAMPT in 200 μL binding buffer for 2 h at 4°C with rotation. For competition experiments, rNAMPT was preincubated with excess free IFITM3 CT peptide (IFITM3-CT) for 30 min at 4°C before addition to the Biotin-IFITM3-CT immobilized beads. After incubation, beads were washed five times with wash buffer and bound proteins were eluted by boiling the beads in SDS sample buffer and analyzed by SDS PAGE followed by immunoblotting using anti-His antibodies.

#### γ-Secretase activity assay

Luciferase reporter-mediated γ-secretase activity assays were conducted as described previously.^75^ Cells were co-transfected with pC99-GVP, pUAS-Luciferase, pβ-galactosidase and either control or constructs. After 24 h, cell extracts were analyzed in luciferase assays following the manufacturer’s instructions (Promega). To control transfection efficiency, the luciferase activity was normalized to β-galactosidase activity.

#### β-Secretase assay

Alkaline phosphatase reporter-mediated β-secretase activity assays were conducted as described below. Cells were co-transfected with pSEAP-APPa-muta and either control or constructs for 24 h pBACE1 was used as a positive control while selective β-secretase inhibitor, LY2886721 (20 mM), was co-treated as a negative control. Harvested medium was heat-inactivated for 1 h at 70 °C to minimize nonspecific enzymatic reaction and then reacted with alkaline phosphatase substrate for 1 h at room temperature. The yellow reaction product was read at 405 nm.

#### α-Secretase assay

α-Secretase assays were performed as described previously.^10^ Cells were incubated for 6 h in serum-free DMEM. The conditioned medium was harvested with cells, and aliquots containing same amount of protein from both the cells and medium concentrated with Amicon Ultra-15 (30 kDa molecular weight cutoff, Millipore) were subjected to SDS-PAGE and western blotting using an anti-sAPPα antibody (anti-6E10). α-Secretase activity was quantified by measuring the sAPPα signal relative to the APP level.

#### *In vitro* AICD generation assay

The conditioned cells were washed with ice-cold PBS and sonicated in buffer A (50 mM HEPES pH 7.4, 150 mM NaCl, 5 mM 1,10phenanthroline monohydrate, 2 mM EDTA, 1 mM PMSF and 1 mM Leupeptin). The nuclear fraction was removed by centrifugation at 1,000 g for 10 min and remaining crude membrane fraction was harvested by further centrifugation at 10,000 g for 15 min. After washing with buffer A, the amount of total protein was quantified by Bradford assay (Bio-Rad). Same amount of protein under resuspended membrane homogenate was incubated for 2–16 h at 37 °C with or without γ-secretase inhibitor, Compound E (Comp.E, 100 mM).

#### Detection of secreted Aβ in culture medium

SH-SY5Y/APPsw or SH-SY5Y/APPslPS1ΔE9 cell lines were transfected with target protein-expressing genes for 48 h. The conditioned culture medium was then harvested and centrifuged at 3,000 g for 15 min to remove cell debris. After debris removal, the conditioned medium was incubated overnight with anti-6E10 or anti-Aβ antibodies. The antibody-protein complexes were pulled down using Protein G beads, and the samples were subjected to SDS-PAGE. For Aβ detection, the acrylamide gel was transferred onto a nitrocellulose (NC) membrane. The transferred membrane was heated in a pressure cooker for 15 min to perform heat-induced epitope retrieval. The NC membrane was then blocked and incubated with anti-6E10 or anti-Aβ antibodies, followed by enhanced chemiluminescence (ECL) detection.

#### Detection of extracellular NAMPT

The conditioned culture medium from various cell types was harvested and centrifuged at 3,000 g for 15 min to remove cell debris. After debris removal, the conditioned medium was incubated overnight with anti-NAMPT or anti-IgG control antibodies. Extracellular NAMPT was pulled down using Protein G beads, and the samples were boiled at 95°C in a heat block before being subjected to SDS-PAGE.

#### Immunoprecipitation of membrane protein

For immunoprecipitation of membrane proteins, harvested cells were fractionated using a fractionation buffer to enrich membrane proteins. The crude membrane fraction was solubilized by adding 0.5% DDM and incubating for 2 h at 4°C. The solubilized fraction was then incubated with target antibodies, and the antibody-protein complexes were pulled down using Protein G beads. The pulled-down samples were subjected to SDS-PAGE for analysis.

#### BN-PAGE

Harvested cells were washed with ice-cold PBS and sonicated in resuspension buffer (50 mM PIPES, pH 7.0, 250 mM sucrose, 1 mM EGTA, and protease inhibitor cocktail). The nuclear fraction was removed by centrifugation at 600*g* for 10 min, and the microsomal fraction was isolated by further centrifugation of the remaining supernatant at 100,000 g for 60 min. Microsomal proteins were solubilized in solubilization buffer (50 mM PIPES, pH 7.0, 250 mM sucrose, 1 mM EGTA, 0.5% dodecylmaltoside, and protease inhibitor cocktail) for 60 min on ice. Solubilized proteins were then separated by BN-PAGE gel (8-10%).

#### SDS-PAGE and western blot analysis

Harvested cells were solubilized in lysis buffer (100 mM HEPES pH 7.4, 150 mM NaCl, 1 mM EDTA, 1% Triton X-100 and protease inhibitor cocktail). The amount of protein was measured by Bradford assay (Bio-Rad) and protein samples were denatured by mixing 2x sampling buffer (60 mM Tris pH 6.8, 2% SDS, 20% glycerol, 10% 2-mercaptoethanol, and 0.04% bromophenol blue) followed by boiling for 10 min at 100C. The denatured protein samples were separated by sodium dodecyl sulfate polyacrylamide gel electrophoresis (SDS-PAGE) using SDS-PAGE gels (10–15% gels) and transferred to polyvinylidene fluoride membrane (PVDF) using semi-dry transfer device (Bio-Rad). Blots were blocked with 3% (w/v) BSA or 5% Milk in TBS-T buffer (25 mM Tris pH 7.5, 150 mM NaCl, and 0.05% Tween 20) for 30 min and incubated with indicated primary antibodies. The probed blots were detected by horseradish peroxidase-conjugated secondary antibodies for ECL analysis.

#### qPCR

Total RNAs were extracted by Trizol method. For reverse transcription, total RNA (1 mg) was reacted with oligo-dT, dNTP, M-MLV reverse transcriptase and M-MLV buffer for 2 h at 42°C. The same amount of cDNA templates (100 ng) was reacted with SYBR green master mixture (Applied biosystems, 4367659) and specific qPCR primers (300 nM) by using qPCR machine (Thermo Fisher Scientific, QuantStudio3).

#### Immunofluorescence

HT22 or MEF cells were incubated on ice with DyLight488-conjugated rNAMPT (1 μg/mL) for 1 h. After washing with PBS, cells were fixed with 4% paraformaldehyde for 15 min at room temperature. Optionally, WGA was applied for 10 min to visualize the plasma membrane. Fixed cells were permeabilized using either 0.1% saponin or 0.1% Triton X-100 in PBS, and subsequently blocked with 1–3% BSA in PBS for 30 min. Cells were incubated overnight at 4°C with primary antibodies against Rab7 (1:200). After washing, appropriate fluorescent secondary antibodies were applied for 2 h at room temperature. Nuclei were counterstained with DAPI, and samples were mounted using an anti-fade mounting medium. Confocal images were acquired using an LSM700 microscope (Carl Zeiss).

To detect plasma membrane-associated rNAMPT, cells were incubated on ice with DyLight488-conjugated rNAMPT (1 μg/mL) for 10–15 min to block endocytosis. After extensive washing with ice-cold PBS containing 1% BSA, cells were fixed in 4% paraformaldehyde. For validation of plasma membrane specificity, a subset of samples was treated with 10 μM methylene blue for 5–10 min at room temperature prior to permeabilization. Cells were subsequently permeabilized with 0.1% saponin, blocked with 1–3% BSA in PBS containing 0.05% Tween 20. Nuclei were counterstained with DAPI, and samples were mounted using an anti-fade mounting medium. Confocal images were acquired using an LSM700 microscope (Carl Zeiss).

#### Immunofluorescence for mouse brain

Mice were intraperitoneally injected with PBS or LPS (0.5 mg/kg) once daily for two consecutive days. Twenty-four hours after the second injection, mice were sacrificed, and brains were collected for frozen sectioning. Brain tissues were sectioned using a cryostat and processed for floating immunofluorescence. Sections were permeabilized with PBS containing 0.3% Triton X-100 for 15 min and then blocked with 3% BSA in PBS for 30 min at room temperature. The sections were incubated with primary antibodies overnight at 4°C. After washing with PBS, sections were incubated with appropriate fluorescence-conjugated secondary antibodies for 1 h at room temperature. Nuclei were counterstained with DAPI, and samples were mounted using an anti-fade mounting medium. Confocal images were acquired using an LSM880 microscope (Carl Zeiss).

#### Single-cell RNA sequencing transcriptomic analysis

Single-cell RNA-sequencing data from whole mouse brains were obtained from the Gene Expression Omnibus (GEO: GSE263094).^44^ Filtered feature–barcode matrices in HDF5 (.h5) format were imported into R (v4.3.3) using Seurat (v5. 5.0). For the present analysis, WT samples from PBS and repeated LPS (LPS ×2) groups were included (WT-PBS, *n* = 2; WT-LPS ×2, *n* = 2). For each sample, a Seurat object was created from the raw count matrix with CreateSeuratObject, retaining genes detected in at least three cells and cells expressing at least 200 features. For each sample, a Seurat object was generated, retaining genes detected in at least three cells and cells with a minimum of 200 detected features. Ambient RNA was corrected using decontX. Cells with fewer than 500 or more than 8,500 detected genes, or with >20% mitochondrial transcripts, were removed. Putative doublets were identified per sample using DoubletFinder (v2.0.6).

For the present analysis, wild-type PBS and repeated LPS (LPS ×2) samples (*n* = 2 per group; 39,097 cells total, 18,016 PBS and 21,081 LPS cells) were subset from the quality-controlled object. The subset was log-normalization (scale factor 10,000) and downstream processing focused on the top 2,000 highly variable genes selected via the variance-stabilizing transformation (vst) method, followed by principal component analysis. Batch correction across samples was performed with Harmony. The first 40 Harmony dimensions were used for UMAP feature plots, dot plots, and heatmaps. Finally, cell type- and condition-specific *Nampt* expression was examined across the annotated clusters.

### Quantification and statistical analysis

ImageJ and image studio software were used for quantification of signal intensity of western blotting and fluorescence intensity of immunostaining. Graph Pad Prism (version 11) software was used for statistical analysis. For statistical analysis of the data from animal models and human tissue samples, the mean ± SEM of the number of subjects was used. For statistical analysis of the data from *in vitro* cell culture experiments, the mean ± SEM of the number of biological replicates was used. For analysis of statistical significance, either Student’s t tests, One-way ANOVA, or two-way ANOVA was used. The significance was defined as *p* values of less than 0.05 and denoted as asterisk (∗*p* < 0.05; ∗∗*p* < 0.01; ∗∗∗*p* < 0.005; ∗∗∗∗*p* < 0.001).
