# Supplementary material for: eNAMPT-IFITM3 axis links neuroinflammation to γ-secretase activation
Source: iScience. 2026 Sep 12;29(10):117496. doi: 10.1016/j.isci.2026.117496 (PMC13590055; doi:10.1016/j.isci.2026.117496)
Supplement: Supplementary file 1 [file mmc1.pdf]

iScience, Volume *29*

Supplemental information

eNAMPT-IFITM3 axis links neuroinflammation to γ-secretase activation

Junho Hyun, Sunmin Jung, Yoona Noh, Yeonsu Cho, Takaomi C. Saido, Jihoon Nah, Ki Woo Kim, and Yong-Keun Jung

SUPPLEMENTARY FIGURES

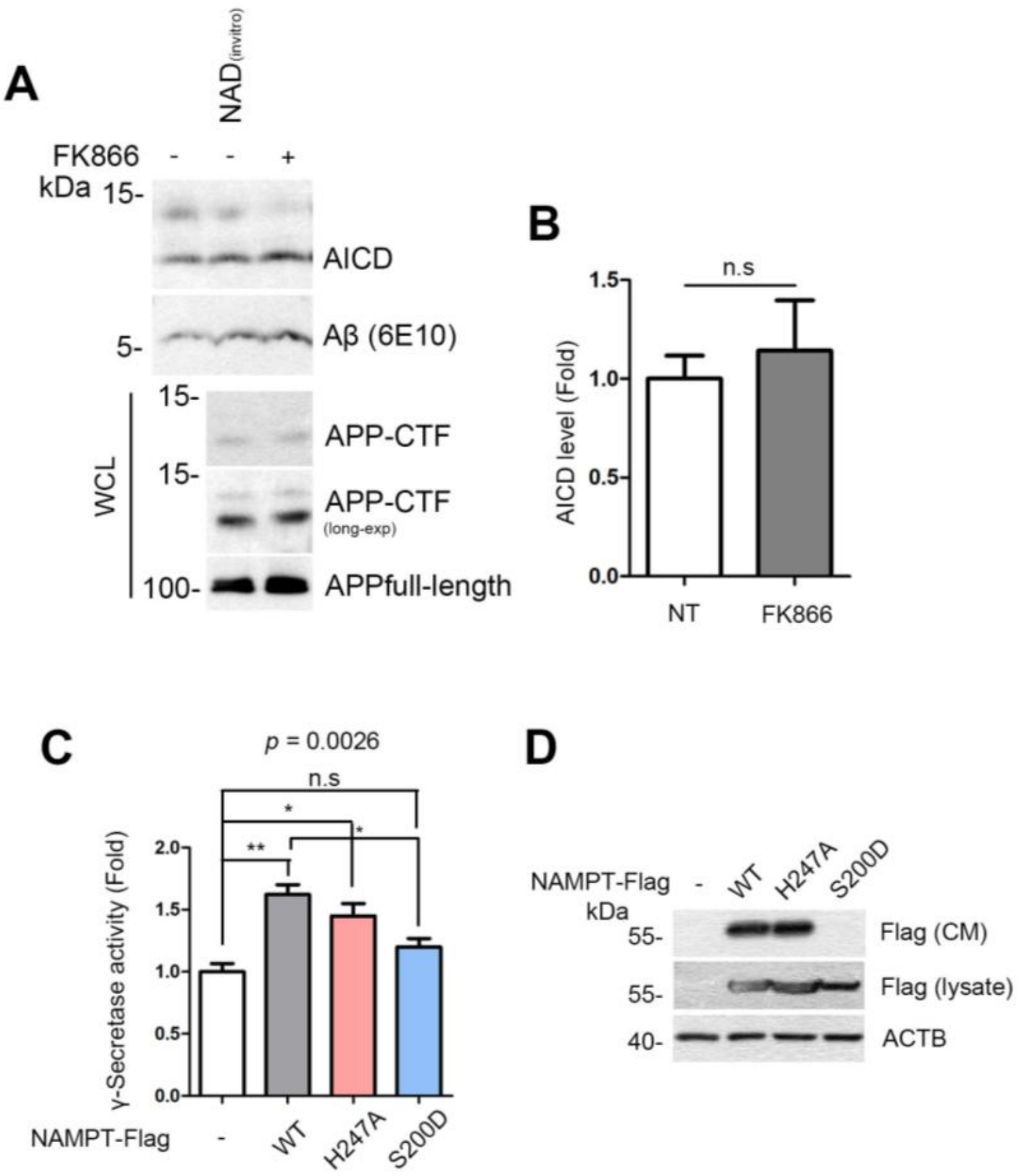

Sup. Figure 1. Enzymatic activity of eNAMPT is not associated with γ-secretase activation.

(**A, B**) HEK293T/APPsw cells were treated with 10 mM FK866 for 24 h and crude membrane fractions were prepared for in vitro AICD generation assay. For NAD treatment group, crude membrane fractions from untreated cells were incubated with 1 mM NAD for 16 h. WCL, whole-cell lysates. (A). The signals of AICD on the blots were quantified using ImageJ. Bars represent mean ± SEM (n = 3), n.s., not significant; Student's t test (B).

(**C, D**) HEK293T cells were co-transfected with a C99-GVP luciferase reporter and NAMPT WT, H247A dominant-negative, or S200D monomeric mutant for 24 h, and γ-secretase activity was measured using a luciferase reporter assay (C). Cell extracts were analyzed by western blotting (D). Bars represent mean ± SEM (n = 3). $*p < 0.05$, $**p < 0.01$, n.s., not significant (One-way ANOVA with post hoc Bonferroni's multiple comparisons test).

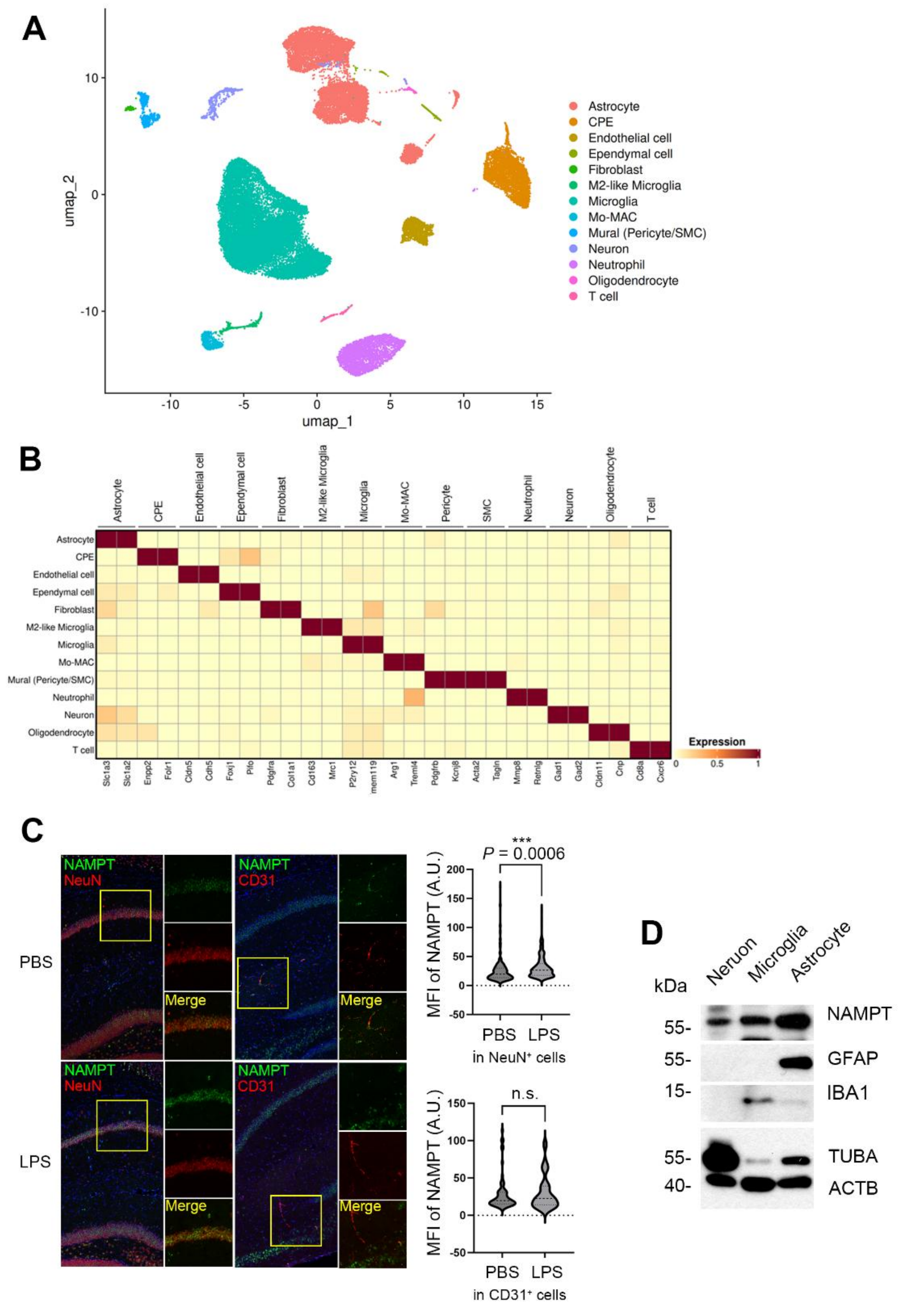

Sup. Figure 2. Characterization of LPS-induced brain NAMPT expression with scRNA-seq and immunofluorescence.

(A) UMAP plots of PBS and LPS-treated (0.8 mg/kg/day for 2 days, i.p.) whole brain cells from GEO dataset GSE263094. After quality control, ambient RNA correction, and doublet removal, 39,097 cells were retained for analysis and visualized after Harmony based integration.

(B) Dot plot of representative canonical marker genes used for cell type annotation of the identified clusters. Clusters were assigned based on differentially expressed genes and established cell type specific markers.

(**C**) Representative images of brain sections immunostained for NAMPT together with cell-type markers, including NeuN for neurons and CD31 for endothelial cells. Mice were i.p. injected with PBS or 0.5 mg/kg LPS daily for two consecutive days, and brain sections were prepared 24 h after the second injection. Scale bar = 100 μm. Quantitative violin plots showing Mean Fluorescence Intensity (MFI) of NAMPT within specific cell marker-labeled areas (NeuN for neurons and CD31 for endothelial cells) between the PBS and LPS group. (Cell numbers: NeuN [PBS = 247, LPS = 245], CD31 [PBS = 68, LPS = 42]). ****$p < 0.0001$ n.s. not significant (Student's t-test).

(**D**) Primary cortical neurons, microglia, and astrocytes (DIV10) were analyzed by Western blotting.

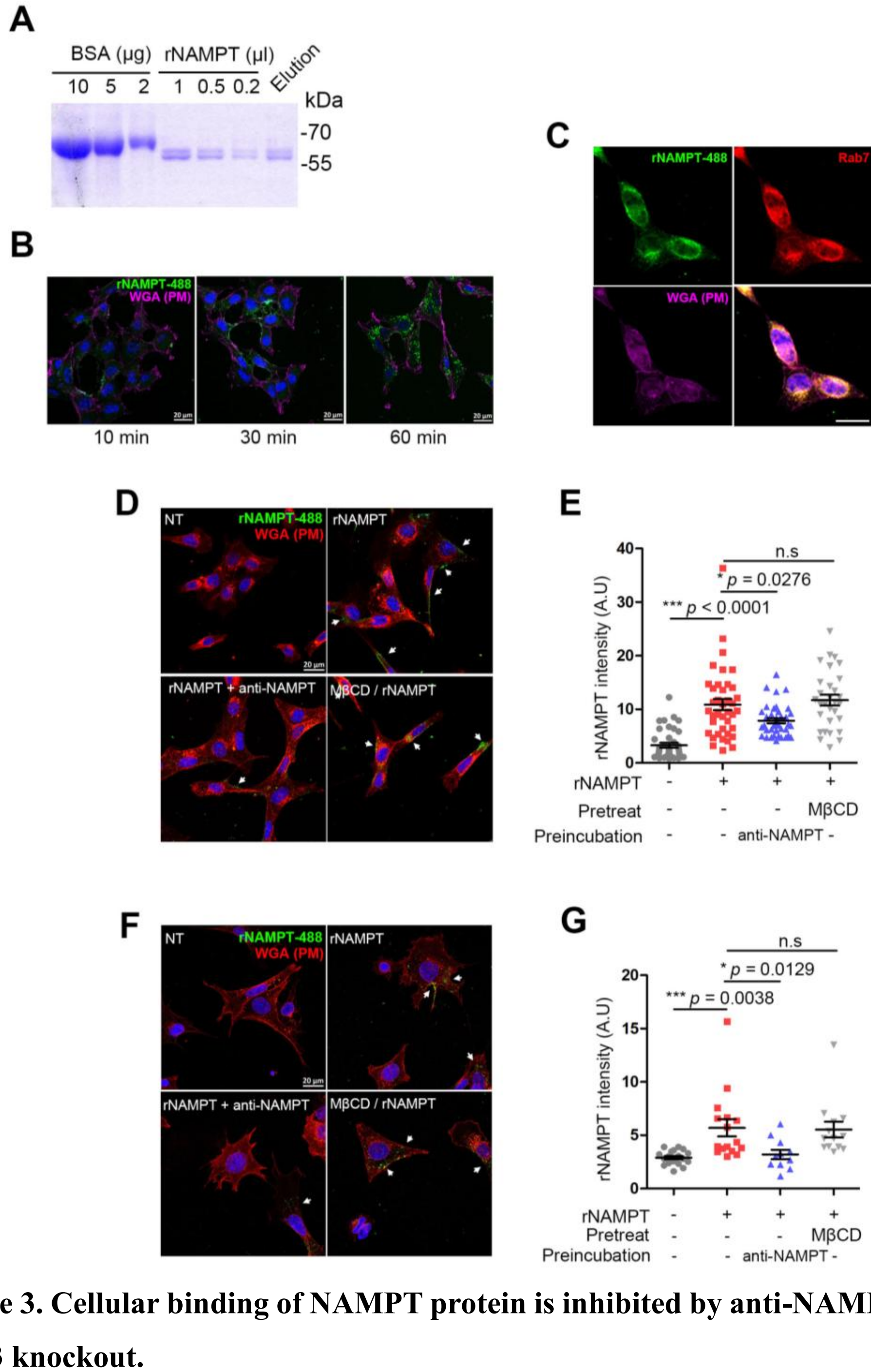

Sup. Figure 3. Cellular binding of NAMPT protein is inhibited by anti-NAMPT antibody or IFITM3 knockout.

(**A**) BL21 Rosetta E coli was transformed with pet28-NAMPT and treated with IPTG for 3 h. His-rNAMPT protein was purified with Ni-NTA beads and stained with Coomassie blue after SDS-PAGE. Purified rNAMPT protein was then subjected to the endotoxin removal process using ToxinCleanic Endotoxin Removal Kit.

(**B**) HT22 cells were treated with 1 μg/mL rNAMPT-488 for 10, 30, and 60 min. Cells were fixed with 4% paraformaldehyde, stained for the plasma membrane using WGA, and then permeabilized with 0.1% saponin. After staining nuclei with Hoechst dye, cells were observed using an LSM700 confocal microscope. Scale bars = 20 μm.

(**C**) After treatment with 1 μg/mL rNAMPT-488 for 1 h, HT22 cells were immunostained with anti-Rab7 antibody (endolysosomal compartment) and Hoechst dye. Images were acquired using LSM700 confocal microscope. Scale bars = 20 μm.

(**D-G**) HT22 cells (D) and Mouse embryonic fibroblasts (MEFs) (F) were pretreated with 5 mM MβCD for 30 min, followed by the treatment with 1 μg/mL rNAMPT-488 or rNAMPT-488 preincubated with anti-NAMPT antibody (1:1, w/w) for 1 h. Cells were fixed with 4% paraformaldehyde, stained for the plasma membrane using WGA, and then permeabilized with 0.1% saponin. After staining nuclei with Hoechst dye, cells were observed using an LSM700 confocal microscope. Arrows indicate cellular rNAMPT-488. Scale bars = 20 μm (D, F). Cellular signal intensities of rNAMPT-488 were quantified using ImageJ (E, G). Bars represent mean ± SEM (cell numbers per group: E; 39, 39, 39, 30 and G; 18, 16, 11, 13), $*p < 0.05$, $***p < 0.005$, n.s., not significant; Student's t test.

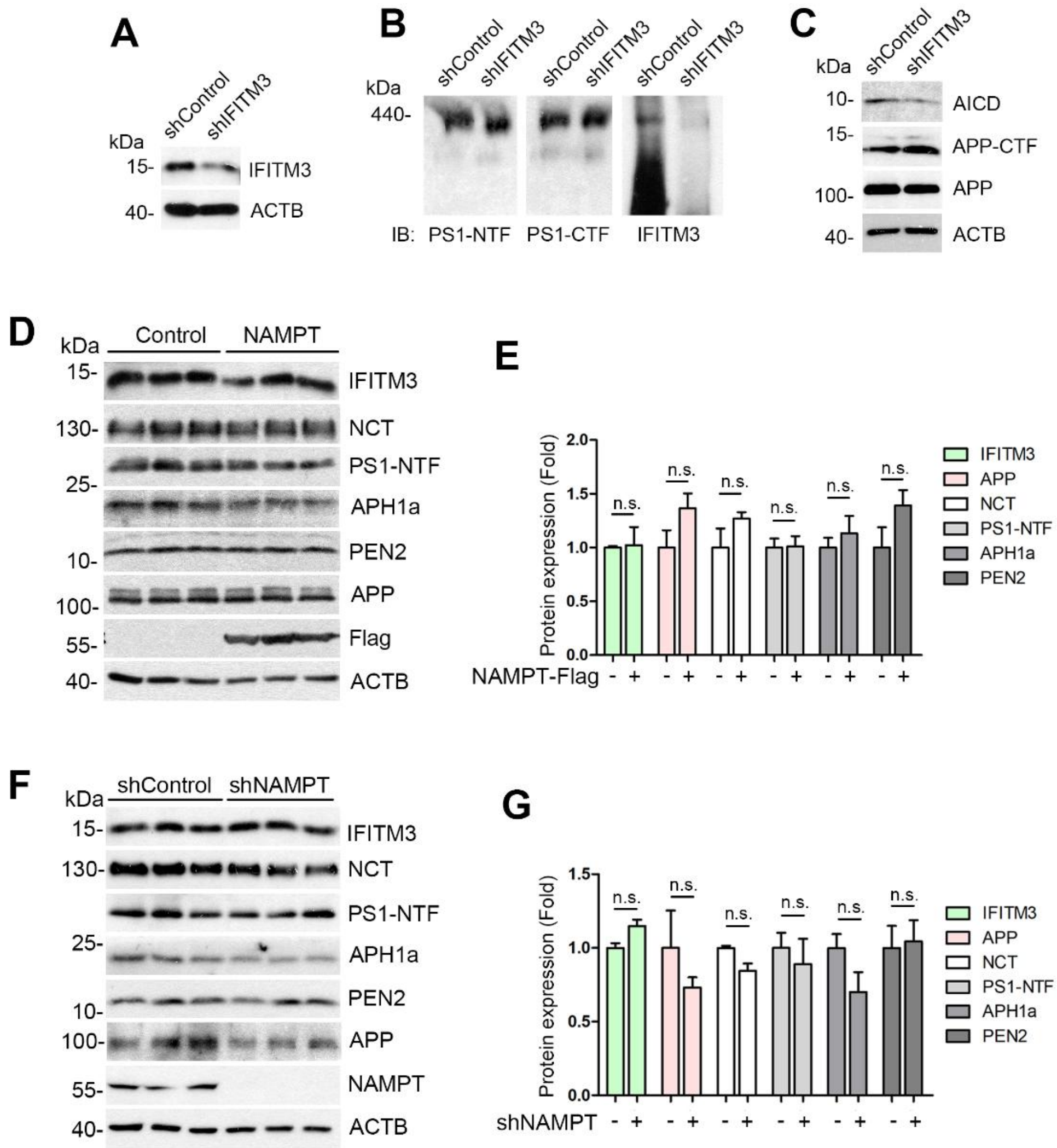

Sup. Figure 4. IFITM3 deficiency disrupts γ-secretase complex assembly and AICD production, and NAMPT modulation doesn't alter γ-secretase components levels.

(**A, B**) HEK293T cells were transfected with shIFITM3 for 48 h and whole cell lysates (WCL) (A) and γ-secretase complexes in crude membrane fractions (B) were analyzed by BN-PAGE and western blotting.

(**C**) SH-SY5Y/APPsl-PS1ΔE9 cells were transfected with control or shIFITM3 for 48 h and crude membrane fractions were subjected to in vitro AICD assay for 2 h.

(**D, E**) SH-SY5Y/APPsl-PS1ΔE9 cells were transfected with NAMPT-Flag for 24 h and analyzed by western blotting (D). The signal of NAMPT, IFITM3, and γ-secretase components on the blots were quantified using ImageJ (E). Bars represent mean ± SEM. n.s., not significant; Student's t test.

(**F, G**) SH-SY5Y cells were transfected with shNAMPT for 48 h and analyzed by western

blotting (F). The signal of NAMPT, IFITM3, and γ-secretase components on the blots were quantified using ImageJ (G). Bars represent mean ± SEM. n.s., not significant; Student's t test

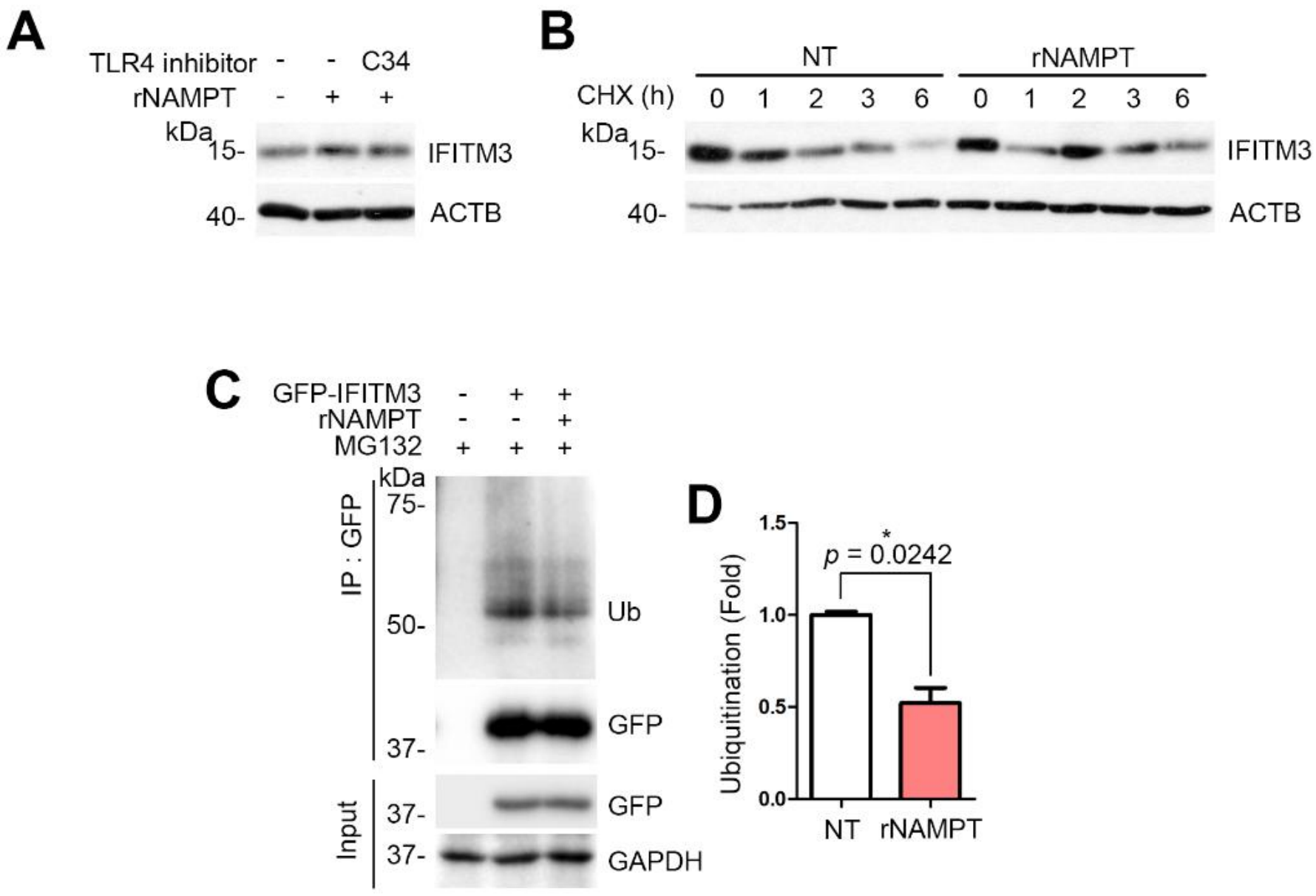

Sup. Figure 5. rNAMPT treatment increases IFITM3 protein stability by reducing its ubiquitination.

(A) HT22 cells were pretreated with 10 μM C34 for 30 min, followed by the treatment with 1 μg/mL rNAMPT protein for an additional 1 h and analyzed by western blotting.

(**B**) HT22 cells were treated with 20 μg/mL cycloheximide (CHX) and with/without 1 μg/mL rNAMPT for the indicated time and analyzed by western blotting.

(C, D) HEK293T cells were transfected with GFP-IFITM3 for 24 h and then incubated in MG132 (10 μM)-containing media with or without rNAMPT (1 μg/ml) for 1h. Cell lysates were immunoprecipitated with GFP-trap and analyzed by western blotting (C). The signals of ubiquitinated GFP-IFITM3 were quantified with ImageJ. Bars represent mean ± SEM. *p < 0.05; Student's t test (D).

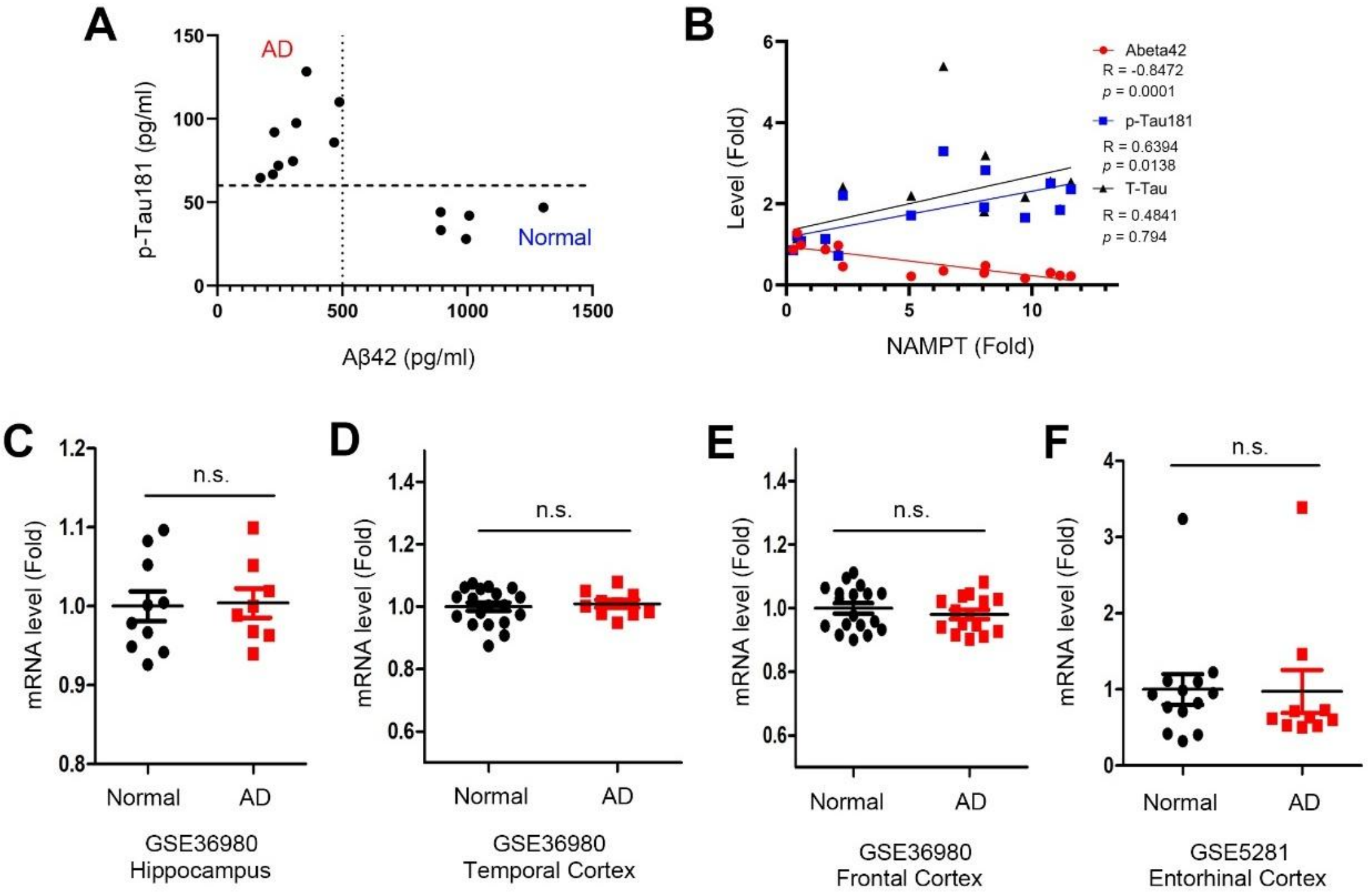

Sup. Figure 6. Validation of AD biomarkers in CSF samples and transcriptomic analysis of NAMPT expression in AD brains.

(**A**) Scatter plot of CSF Aβ42 and p-Tau181 concentrations from AD patients and non-AD controls. Dashed lines indicate commonly used diagnostic thresholds for Aβ42 (500 pg/ml) and p-Tau181 (60 pg/ml), demonstrating clear separation between AD and normal samples.

(**B**) Correlation analysis between NAMPT levels and CSF AD biomarkers, including Aβ42 (red), phosphorylated Tau (p-Tau181, blue), and total Tau (T-Tau, black).

(**C-F**) Relative levels of NAMPT mRNA in the hippocampus (C), temporal cortex (D), frontal cortex (E), and entorhinal cortex (F) of patients with AD and Non-AD. Data were extracted from GSE36980 (C-E) and GSE5281 (F). Bars represent mean ± SEM. n.s., not significant; Student's t test.

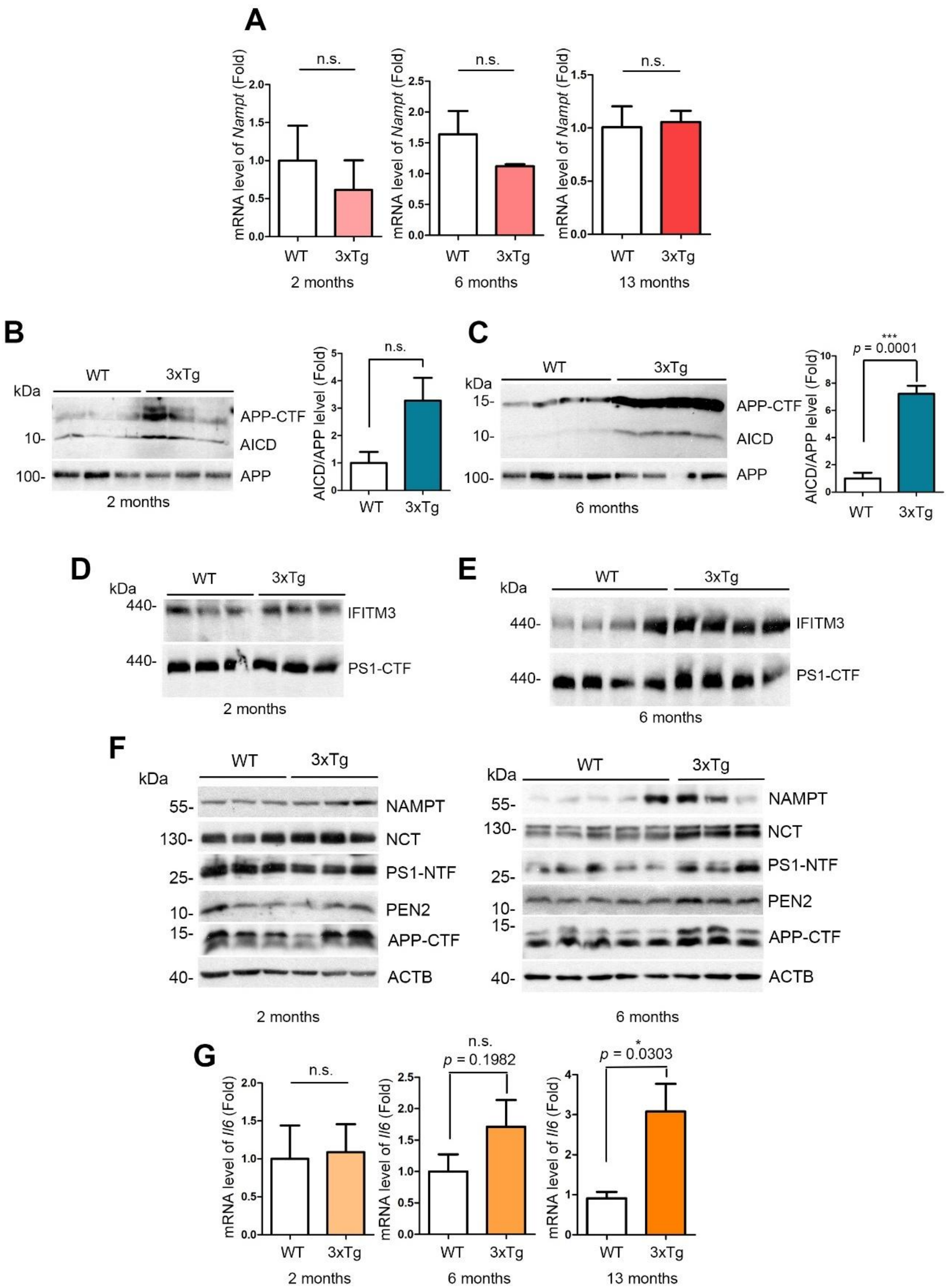

A
n.s.
mRNA level of Nampt (Fold)
WT 3xTg
2 months
6 months
13 months
B
WT 3xTg
kDa
APP-CTF
10- AICD
100- APP
2 months
AICD/APP lelvel (Fold)
C
*** p = 0.0001
15- APP-CTF
10- AICD
100- APP
6 months
D
440- IFITM3
440- PS1-CTF
2 months
E
6 months
F
55- NAMPT
130- NCT
25- PS1-NTF
10- PEN2
15- APP-CTF
40- ACTB
2 months
6 months
G
mRNA level of Il6 (Fold)
n.s. p = 0.1982
* p = 0.0303
13 months

Sup. Figure 7. NAMPT protein, not mRNA, is elevated in the brains of 3xTg mice.

(**A)** Levels of NAMPT and γ-secretase components were assessed by western blotting in the cortical extracts of 2-month (Left) and 6-month (Right)-old 3xTg mice.

(**B, C**) Total RNAs were isolated from the cortex of 2-month, 6-month, and 13-month-old 3xTg mice by Trizol. The mRNA levels of NAMPT (B) and IL6 (C) were analyzed by QuantStudio qPCR. Bars represent the mean ± SEM [n; WT (2-month-old) = 3, 3xTg (2-month-old) = 4, WT (6-month-old) = 4, 3xTg (6-month-old) = 3, WT (13-month-old) = 4, 3xTg (13-month-old) = 5]. n.s., not significant; Student’s t test.

(**D, E**) Crude membrane fractions from WT and 3xTg (2 and 6-month) cortex were subjected to in vitro AICD generation assay, followed by western blotting. The signals of AICD on the blots were quantified using ImageJ. Bars represent mean ± SEM. [(D, 2-month), (E, 6-month)]. ***$p < 0.005$, n.s., not significant; Student's t test.

(**F, G**) Crude membrane fractions from WT and 3xTg (2 and 6-month) cortex solubilized with 0.5% DDM were analyzed with BN-PAGE and western blotting [(F, 2-month), (G, 6-month)].

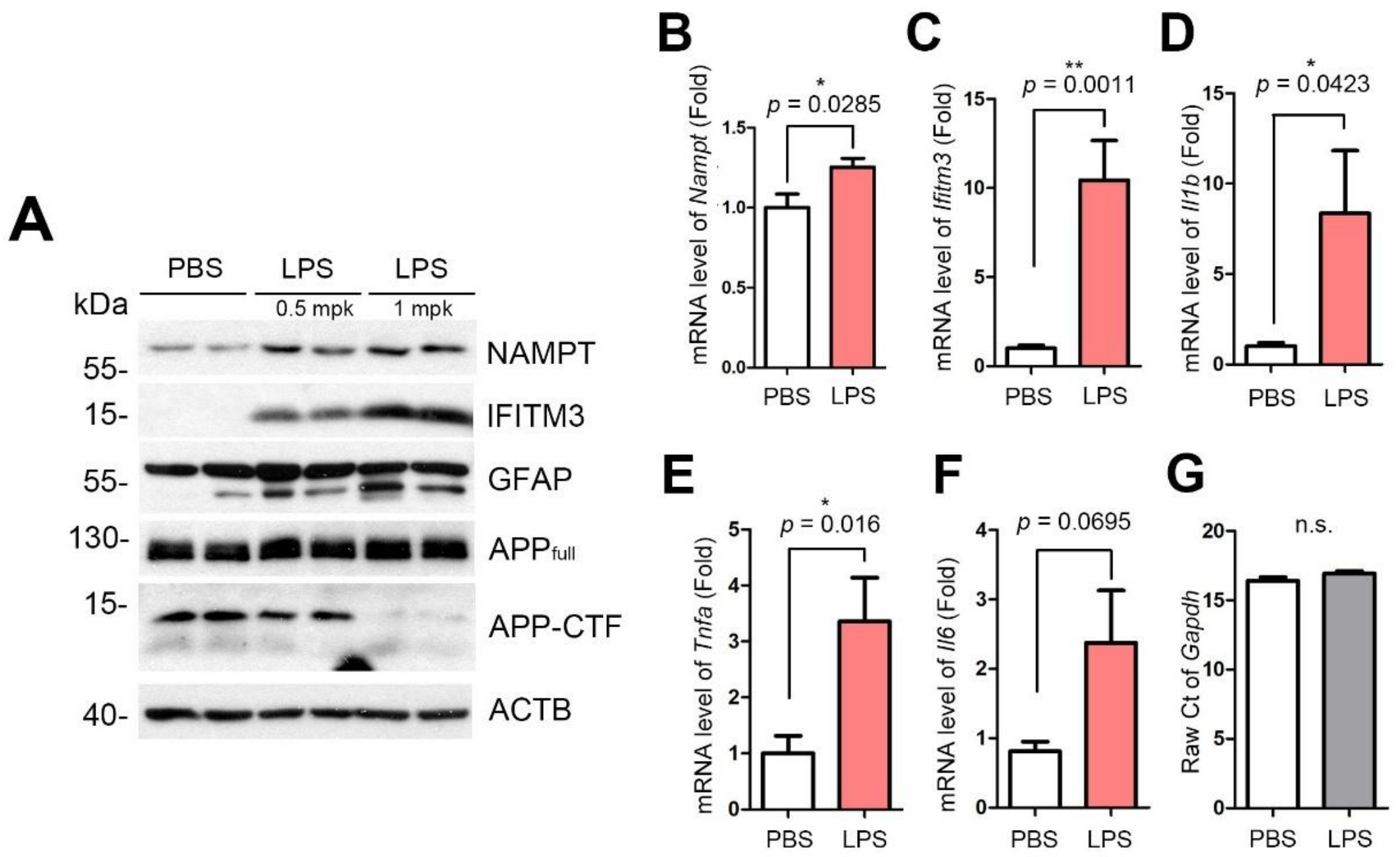

Sup. Figure 8. Acute inflammation induces NAMPT and IFITM3 mRNAs in the mouse brain.

(**A**) Three-month-old C57BL6 mice were daily and intraperitoneally injected with 0.5 mg/kg and 1 mg/kg LPS for 2 days. Mouse cortex was analyzed by western blotting.

(**B-G**) Three-month-old C57BL/6 mice were daily and intraperitoneally injected with 0.5 mg/kg and 1 mg/kg LPS for 2 days. Total RNAs were extracted from mouse cortex with Trizol reagent and analyzed by RT-qPCR (Quant-studio 3) for Nampt (B), Iifitm3 (C), Il1β (D), Tnfα (E), and Il6 (F). Gapdh is used for reference gene (G). Bars represent mean ± SEM. (n; PBS = 6-7, LPS = 6-7). *p < 0.05, **p < 0.01; Student's t test.

Table S1. CSF samples information

#	Dignosis	Ab42 (pg/ml)	T-tau (pg/ml)	P-tau181 (pg/ml)	Amlyoid PET reading
1	Community based normal	892.5	224.8	44.2	Negative
2	Community based normal	994.5	201.8	28.1	Negative
3	Subjective memory impairment	1006.8	275.6	42.1	Negative
4	Subjective memory impairment	1030.8	269	46.9	Negative
5	Subjective memory impairment	893.7	202.1	33.3	Negative
6	LOAD	227.5	595.2	92	Positive
7	EOAD	315.4	601.6	97.6	Positive
8	LOAD	243.6	442.6	72.1	Positive
9	EOAD	488.1	750.9	110.2	Positive
10	EOAD	172	508.9	64.7	Positive
11	EOAD	302.3	426.7	74.7	Positive
12	LOAD	356.4	1265.7	128.4	Positive
13	LOAD	222.3	516.6	66.8	Positive
14	EOAD	467	567.2	85.9	Positive

Table S2. Human brain samples information

Group	Sample ID	Sex	Age	PMI	Neuropathological Diagnosis
**Control**	BB18-09	M	56	9	Brain within normal limits
	BB19-11	M	87	9	Primary age related tauopathy (PART)
	BB21-09	M	60	7.5	PART, Braak stage I
	BB19-15	M	56	6.5	c/w alcoholic brain disease
	BB19-25	F	41	23.5	Mild atherosclerosis (PART context)
	BB22-05	F	82	2.5	PART, Braak stage III
	BB24-08	M	59	6	PART, minimal pTau in transentorhinal cortex
**AD**	BB17-02	F	75	N/A	Clinically esophageal squamous cell carcinoma
	BB18-03	M	81	17	AD with severe reactive gliosis
	BB18-13	F	93	13	AD
	BB21-03	M	75	17	AD, Braak stage VI, CERAD 3
	BB18-02	F	75	5	AD
	BB18-07	M	83	29	AD
	BB20-10	M	83	18	AD
